# The MedEdPORTAL Infinity Mirror: Conducting an Interactive Workshop on How to Develop an Educational Summary Report for MedEdPORTAL

**DOI:** 10.15766/mep_2374-8265.11197

**Published:** 2021-10-22

**Authors:** Richard L. Sabina, Gordon L. Woods, Hannah Turner, Emine Abali, Jana M. Simmons, Grace C. Huang

**Affiliations:** 1 Adjunct Professor, Department of Foundational Sciences, Oakland University William Beaumont School of Medicine; 2 Associate Professor of Medicine and College Mentor, Texas Tech University Health Sciences Center at El Paso, Paul L. Foster School of Medicine; 3 Senior Staff Editor, *MedEdPORTAL*, Association of American Medical Colleges; 4 Assistant Dean for Basic Science Curriculum, CUNY School of Medicine; 5 Associate Professor, Michigan State University College of Human Medicine; 6 Dean for Faculty Affairs, Harvard Medical School

**Keywords:** Faculty Development, Leadership Development/Skills, Mentoring/Coaching, Publishing/Scholarship

## Abstract

**Introduction:**

*MedEdPORTAL* is an open-access journal for health professions educators to publish their educational activities. The Educational Summary Report (ESR) is the manuscript that represents scholarly expression of those activities, aligned with Glassick's criteria for scholarship; however, prospective authors face challenges in writing ESRs, which can lead to rejection.

**Methods:**

We developed a conference workshop to teach health professions educators how to write an ESR by reviewing a sample ESR in small groups. The workshop began with a didactic on best practices in crafting each section of an ESR. We then divided participants into small groups to review an assigned section of a sample ESR using a reviewer's checklist and completing a templated flip chart. Each small group then reported out in a large-group discussion. A conference evaluation was distributed online to solicit perceptions of the workshop's effectiveness.

**Results:**

The 90-minute workshop was presented by separate teams of two facilitators at three national conferences. Approximately 35 participants attended the first workshop, and 50 attended the second and third workshops. Survey feedback from 19 respondents (38%) to the evaluation survey at the third workshop was representative of the previous two iterations and demonstrated that workshop content and materials were helpful.

**Discussion:**

A workshop enabling educators to serve as group peer reviewers of a sample ESR for a *MedEdPORTAL* submission was well received. Associate editors, faculty mentors, and other experienced faculty development leaders can use these materials to support future authors in submitting to *MedEdPORTAL* while providing opportunities for national presentations.

## Educational Objectives

By the end of this activity, learners will be able to:

1. Compare and contrast the Educational Summary Report (ESR) in *MedEdPORTAL* with a traditional manuscript.
2. Describe each part of the ESR and how well-written components adhere to standards for scholarship.
3. Identify potential pitfalls in each section of an ESR.
4. Describe best writing practices in developing an ESR.

## Introduction

For many years, the expansion of medical schools and university hospitals has driven the recruitment of large numbers of clinician-educators to support teaching and patient care.^1^ Traditionally, universities have based academic promotion on research productivity, not teaching excellence.^2^ As a result, many clinician-educators have failed to advance in academic rank, and turnover in these positions has been high.^3,4^ Responding to this, leaders in medical education have advocated that academia formally recognize the scholarship of teaching,^5^ provide training in educational scholarship to clinician-educators,^6–8^ and establish criteria for academic promotion of clinician-educators.^9,10^

Essential to the support of clinician-educators has been the creation of peer-reviewed venues where they could publish educational scholarship. Responding to this need, in 2005, the Association of American Medical Colleges established *MedEdPORTAL: The Journal of Teaching and Learning Resources.*^11^ In 2016, *MedEdPORTAL* adopted a formal submission format, the Educational Summary Report (ESR), to align the reporting of educational activities with broadly accepted standards of scholarship.^12^ The ESR incorporates the standard IMRAD (introduction, methods, results, and discussion) framework that has been adopted by academic journals worldwide.^13^

Writing manuscripts has historically been a source of difficulty for authors.^14^ In the broader medical education literature, reasons for rejection by editors include a vague statement of purpose, incomplete description of methods, underreporting of results, and unclear interpretation of the findings.^15–17^ Consequently, publications on manuscript writing are popular.^18,19^ Similarly, authors wishing to submit to *MedEdPORTAL* have been challenged by the process of writing ESRs that meet scholarly standards, and weak ESRs have been a reason that submissions have either struggled in the peer review process or been a source of frustration for associate editors (unpublished data).

A number of publications in *MedEdPORTAL* have similarly described how to prepare submissions to *MedEdPORTAL.*^20,21^ However, none have focused specifically on the ESR, which is a relatively new submission requirement. Additionally, none of these publications have been authored from the perspective of editors who routinely read and critique ESRs.

To address the needs of educators wishing to publish their work in *MedEdPORTAL,* we developed a workshop to teach prospective authors the underlying structure of the ESR. In terms of instructional methodologies, the workshop format consisted of a short didactic followed by a small-group, guided critique of an ESR; of note, peer review of draft manuscripts is an established approach to learning scholarly writing skills.^22–24^ Below, we review our experience with three presentations of this workshop over the course of 3 years.

## Methods

### Needs Assessment

We conducted an informal needs assessment that led to the development of this workshop. We have anecdotally observed, since instituting the ESR in 2016, that authors struggle with writing this document in a way that meets scholarly standards. *MedEdPORTAL* faculty mentors,^25^ who are volunteer faculty who help prospective authors prepare work for submission to *MedEdPORTAL*, have expressed that writing the ESR is a challenge. Additionally, one initiative to improve the quality of ESRs—a checklist of ESR requirements available on the *MedEdPORTAL* website^26^—seemed to have variable adherence. Lastly, nearly all submissions to *MedEdPORTAL* garnered comments from peer reviewers, associate editors, or the editor-in-chief on how the ESR was written, signaling an opportunity for development.

### Workshop Development

We designed a workshop intended to teach prospective *MedEdPORTAL* authors about best practices in writing the ESR from the perspective of a peer reviewer. Instruction centered on a structured exercise of critiquing a sample ESR in small groups. Building on lessons learned from the first two iterations, namely, the 2018 Southern Group on Educational Affairs Regional Meeting^27^ and the 2019 International Conference of the Association of Biochemistry Educators,^28^ we developed an outline for a third workshop, iteratively revised the outline over email, and submitted the workshop proposal to the 2019 Annual International Association of Medical Science Educators Meeting.^29^

After receiving notice of the acceptance of our workshop, we discussed specifics of workshop execution over the course of three meetings. Designing the workshop to foster credibility, facilitate peer learning, and promote the use of higher-order cognitive skills, we planned to have participants work in small groups as they performed a structured critique of an authentic example ESR.

### Facilitators

Each workshop was facilitated by teams of two facilitators who had significant experience as peer reviewers and reviewing ESRs specifically. Overall guidelines for preparing for the workshop are included in Appendix A.

### Identification of an ESR for Review

The centerpiece of the workshop was a facilitated group exercise reviewing a draft ESR. To identify an ESR to use during each workshop, we reached out to key leaders within the target organizations. We also sent emails to recruit prospective *MedEdPORTAL* authors to submit ESRs for review. After identifying a volunteer, we connected with the prospective authors 2 months in advance to offer guidance and resources while they were drafting their ESR. We set expectations for having a draft ESR 3 weeks prior to the workshop. We relayed feedback to the authors 2 weeks in advance of the workshop regarding missing elements (e.g., more references, including structured abstract, alignment with worksheet), and 1 week prior to the workshop, we finalized a draft ESR for distribution.

An optional approach to finding an ESR to review during the workshop would be to use an already published ESR from a *MedEdPORTAL* publication. We include guidelines for choosing a published ESR in Appendix A. While these will be by nature more aligned with scholarly writing principles, even published articles can be improved upon.

### Workshop Implementation

The summary of a 90-minute workshop is listed below, with only 80 minutes specifically assigned, to allow buffer time for late starts and longer-than-expected discussions.

• Introduction and didactic: 20 minutes.
• Small-group exercise: 30 minutes.
• Report-out on each of the ESR sections: 20 minutes.
• Wrap-up: 10 minutes.
• Distribution of take-home materials: at the end of the workshop.

During the didactic, we first used the slides and talking points in Appendix B to review aspects of the ESR and how they are comparable to and differ from traditional research manuscripts. We invited participants to ask questions throughout the presentation to foster a safe learning environment and recapitulation of particular points that may not have been clear.

We then divided the room into groups of four to eight participants, assigning each group to be responsible for one of the ESR sections. Multiples of five groups are ideal to cover each of the sections of (1) abstract/objectives, (2) introduction, (3) methods, (4) results, and (5) discussion, but if necessary, abstract/objectives and introduction can be combined to permit four groups. We instructed all participants to read through the ESR to be reviewed, allotting 5 minutes. We then asked the groups to identify individuals for each of three different roles—discussion moderator, timekeeper, and scribe. More than one of these roles could be performed by the same individual, depending on group size. We asked each group to spend 20 minutes as a group critiquing the ESR section by section, using the ESR review checklist (Appendix C) to guide feedback on the ESR. We then asked the group members to spend the last 5 minutes helping the scribe complete a templated flip chart on their assigned ESR section (see photos in Appendix A) for reporting out to the large group. Virtual versions of this workshop could take advantage of videoconferencing functionality for creating breakout rooms and using Google Drive for scribing the groups' insights, and we elaborate on these tips in Appendix A.

We then moderated a large-group report-out of feedback on the ESR section by section, in the following order according to how groups were assigned: abstract/objectives, introduction, methods, results, and discussion. To keep the report-out concise, we prompted group reporters to identify take-home points about deficiencies in the sample ESR, rather than summarizing the group discussion. The guidance language shown in Table 1 helped keep the report-out high yield for the audience, rather than redundant.

**Table 1.**
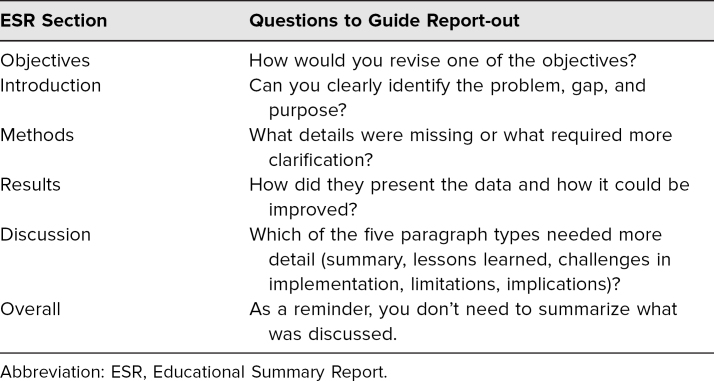
ESR Guidance by Section

We concluded the workshop with a summary of take-home points from the large-group discussion and left 10 minutes at the end for questions. We distributed the ESR worksheet (Appendix D), a handout that provided prompting questions on each section of the ESR, as an additional resource for future submissions to *MedEdPORTAL.*

### Evaluation

The workshop was presented by separate teams of two facilitators at three medical education conferences, but it was formally evaluated only during the third workshop. The conference organizers distributed to all attendees an online standardized survey, one not designed by the authors of this work, soliciting feedback on specific workshops attended (Appendix E). We have also included in Appendix E a suggested revised evaluation that is better aligned with the objectives of the workshop. The survey included six Likert-type questions and one question for open-ended comments. We tabulated the results by dichotomizing the Likert-scale responses into strongly agree/agree versus other responses and reported the frequency of responses with a positive valence. We collated the comments and categorized them by predominant themes.

## Results

Approximately 35 participants attended the first workshop, and there were 50 participants at both the second and third workshops, for a total of about 135 participants. The conferences targeted health professions educators across the spectrum of medical educators, but we did not collect demographic information from the participants specifically. Of those surveyed for the third workshop, 19 completed the online evaluation survey (response rate: 38%). Evaluation results are summarized in Table 2 and attest to the relevance of the content and the helpfulness of the presenters. The handout materials were rated as less effective.

**Table 2.**
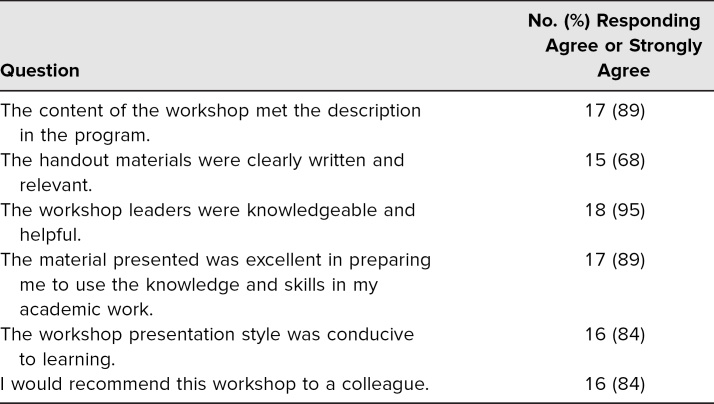
Evaluation Survey Responses (*N* = 19)

We received 10 responses to our open-ended question soliciting comments; these we categorized into three themes:

• That choosing an ESR was key to the workshop and may have improved the experience had it already been a published manuscript:
  ○ “Criticizing and evaluating a poorly written manuscript did not work for me. I think looking at an excellent paper would have worked much better. We could have followed the lead in a positive way, instead of slamming someone's research.”
  ○ “I'd appreciate it if the session focused more on the specifics of MedEd portal, maybe by providing a variety of successful examples that have been accepted, rather than by spending majority of the time on a single draft that has not been accepted yet.”
  ○ “Make it clear from the get go that it hasn't been accepted and that we are to act as the reviewers.”
• That in general the workshop was considered well designed:
  ○ “Could have used a little more time since there were a number of questions early on, but the presentation was excellent and valuable. I look forward to making my first MedEdPortal submission!”
  ○ “Great topic speakers organized, yet flexible and able to adapt to the needs of the audience great materials provided to make this an active session—good balance between didactic material and hands-on activities.”
  ○ “The exercise was great, just the right amount of time.”
• That similarly, the workshop was useful, though this was not universally perceived:
  ○ “Thank you so much for this helpful session!”
  ○ “Great information and useful activity.”
  ○ “By the end, I think I gain knowledge to help me with a future MedEdPortal submission, for which I'm grateful.”
  ○ “I still feel like I'll now have to go to MedEd portal and study closely the accepted submissions posted there in order to get a feel for what my own submission should look like, which I hoped this workshop would provide me with.”

## Discussion

To help prospective authors prepare submissions to *MedEdPORTAL*, we developed an interactive workshop on the ESR and implemented it at three national medical education conferences. Our evaluation of the third workshop showed that the session was well received and was considered practical and useful.

The ESR worksheet (Appendix D) directed participants to review specific aspects of the sample ESR that were identified by Bordage,^15^ Meyer and colleagues,^16^ and Norman^17^ as frequent reasons for rejection. These include the following:

• Statement of the problem,^15–17^
• Review of the literature,^15–17^
• Statement of purpose,^15,17^
• Complete description of methods,^15,16^
• Results appropriately organized and presented,^15,16^
• Relevance and generalizability,^15–17^ and
• Forthright interpretation of results.^15–17^

Participants reported appreciating the opportunity to review a prepublication ESR to observe how a scholarly work would require refinement prior to publication. They also enjoyed serving as group peer reviewers during the exercise, which allowed them to learn from each other how each viewed the elements of an ESR.

We learned that some sections of the ESR are easier to generate guiding principles for than others. The introduction, for instance, though more challenging because it is based on prose, is more straightforward to teach using the adaptation of frameworks like Lingard's problem, gap, hook.^30^ Additionally, introductions tend to be shorter than other sections of the ESR. The discussion section similarly has subsections that are well defined by the ESR template in *MedEdPORTAL*'s author instructions^26^ and is in general focused on personal reflection. We found that the methods and results (to some extent) are so individualized that critiques of these sections are hard to generalize as principles to one's own projects. For these sections, we reinforced the importance of adopting the viewpoint of a future reader hoping to implement the activity in their own institution.

We also discovered that choosing the right ESR for review was essential. The example we used for the third iteration was nine pages long and required more time to read and critique than a typical ESR of four to six pages. In addition to the challenge of identifying an ideal ESR, it took a significant amount of time to find an author willing to prepare an ESR within the time line of the workshop. We required several rounds of emails to colleagues before an opportunity materialized. Additionally, advance preparation time of several weeks was necessary, as we communicated multiple times with the authoring team of the ESR in asking them to include additional elements. Lastly, it was challenging to fully understand the educational activity described in the ESR without access to the appendices, which we had purposefully withheld to streamline the workshop. Evaluation results also revealed that choosing a higher-quality ESR would have exposed participants to well-written sections, therefore modeling strong scholarly writing practices.

The most prominent limitation to our work is the paucity of outcomes. The first two iterations were not systematically evaluated, which was a missed opportunity for continuous improvement. For our third iteration, conference organizers used a workshop survey generalizable to all workshops at the conference, and thus, our evaluation was not geared specifically to the objectives, nor did it meaningfully assess transfer of understanding into behavior. We could also have improved our response rate by supplying a paper version of our evaluation at the end of the workshop. We did not seek the identities of any of the participants at our three iterations to follow up on whether they ended up submitting *MedEdPORTAL* manuscripts and, even more valuably, whether such submissions resulted in positive editorial outcomes. Our evaluation also represented the insights of only a subset of all participants of these workshops; selection bias may have played a role in receiving mostly positive evaluations of our workshop.

Another limitation of our work relates to the setting in which this workshop is replicated. The background of the audience will affect its receptivity to scholarly writing principles; more novice participants may struggle with nonintuitive writing standards such as using the first-person, active voice. Additionally, we did not have sufficient time in a onetime workshop to allow participants to apply these principles to their own writing.

A workshop focused on identifying problems in ESRs is one potential step towards assisting future authors in their own submission preparations for *MedEdPORTAL.* Future iterations of this workshop should provide a polished ESR for review and also allow opportunities for participants to apply the principles and skills conferred to their own projects, perhaps resulting in a draft ESR for their own future submissions. Furthermore, having groups of two or three participants exchange contact information may allow them to serve as accountability partners to each other for future submissions.

## Appendices


Guidance for Facilitators.docxMEP ESR Workshop Slides.pptxEvaluating a Sample ESR.docxESR Worksheet.docxWorkshop Evaluation.docx
*All appendices are peer reviewed as integral parts of the Original Publication.*
