# Supplementary material for: The MedEdPORTAL Infinity Mirror: Conducting an Interactive Workshop on How to Develop an Educational Summary Report for MedEdPORTAL
Source: MedEdPORTAL. 2021 Oct 22;17:11197. doi: 10.15766/mep_2374-8265.11197 (PMC8552417; doi:10.15766/mep_2374-8265.11197)
Supplement: Supplementary file 1 — Guidance for Facilitators.docxMEP ESR Workshop Slides.pptxEvaluating a Sample ESR.docxESR Worksheet.docxWorkshop Evaluation.docx [file mep_2374-8265.11197-s001.zip › B. MEP ESR Workshop Slides.pptx]

## Slide 1
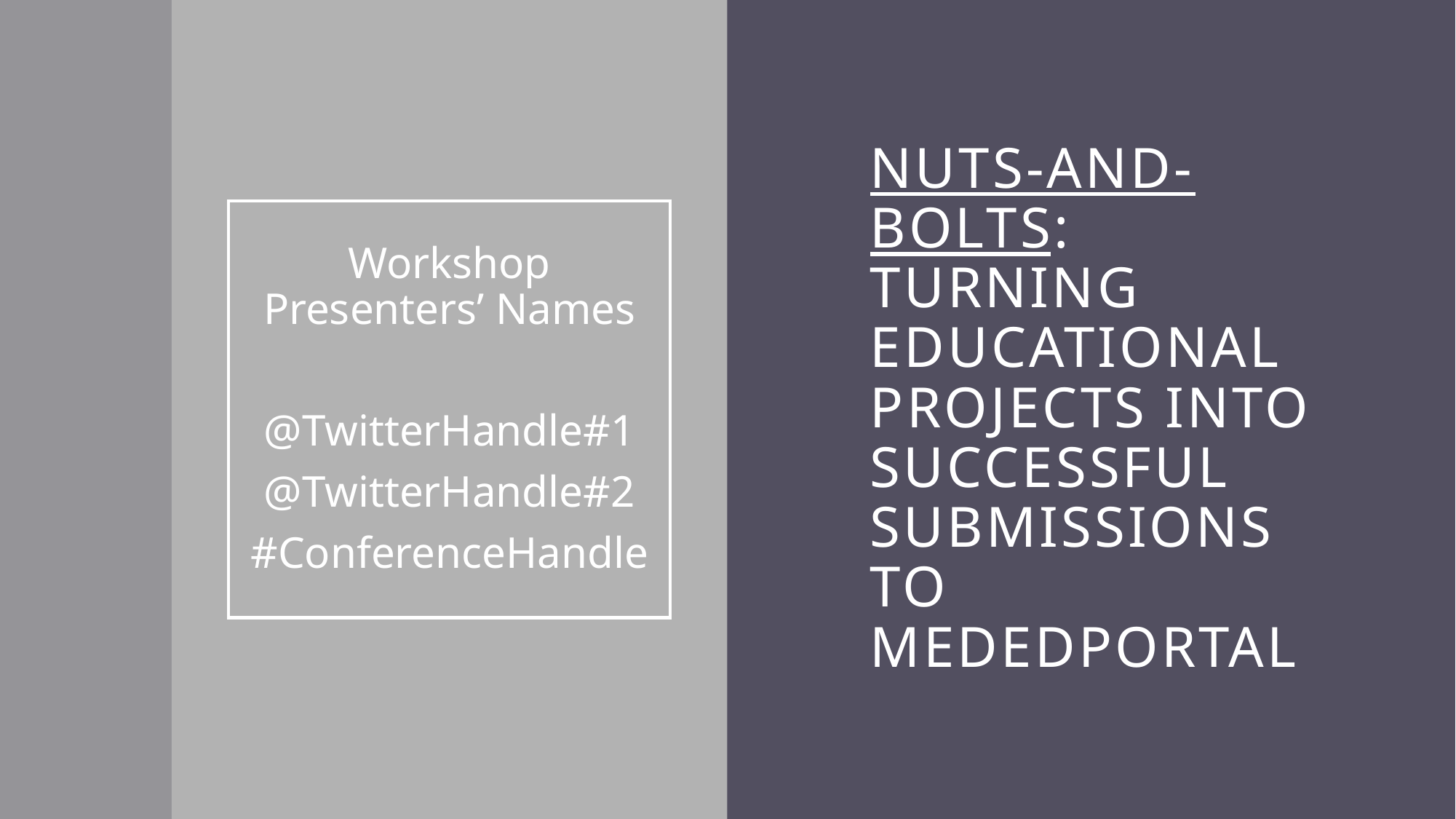

# Nuts-and-Bolts: Turning Educational Projects into Successful Submissions to MedEdPORTAL
Workshop Presenters’ Names
@TwitterHandle#1
@TwitterHandle#2
#ConferenceHandle

## Slide 2
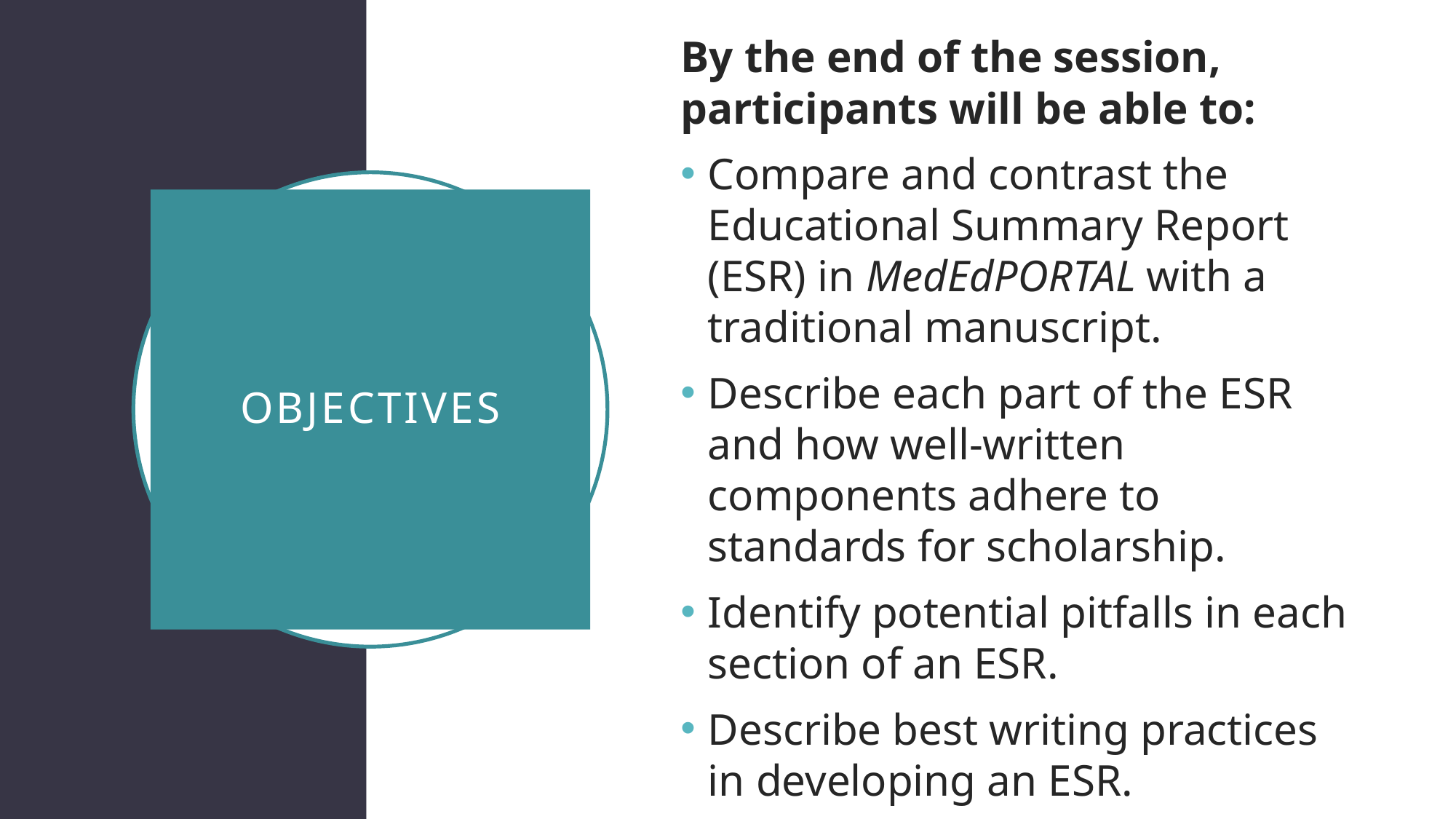

By the end of the session, participants will be able to:
Compare and contrast the Educational Summary Report (ESR) in MedEdPORTAL with a traditional manuscript.
Describe each part of the ESR and how well-written components adhere to standards for scholarship.
Identify potential pitfalls in each section of an ESR.
Describe best writing practices in developing an ESR.
# Objectives

## Slide 3
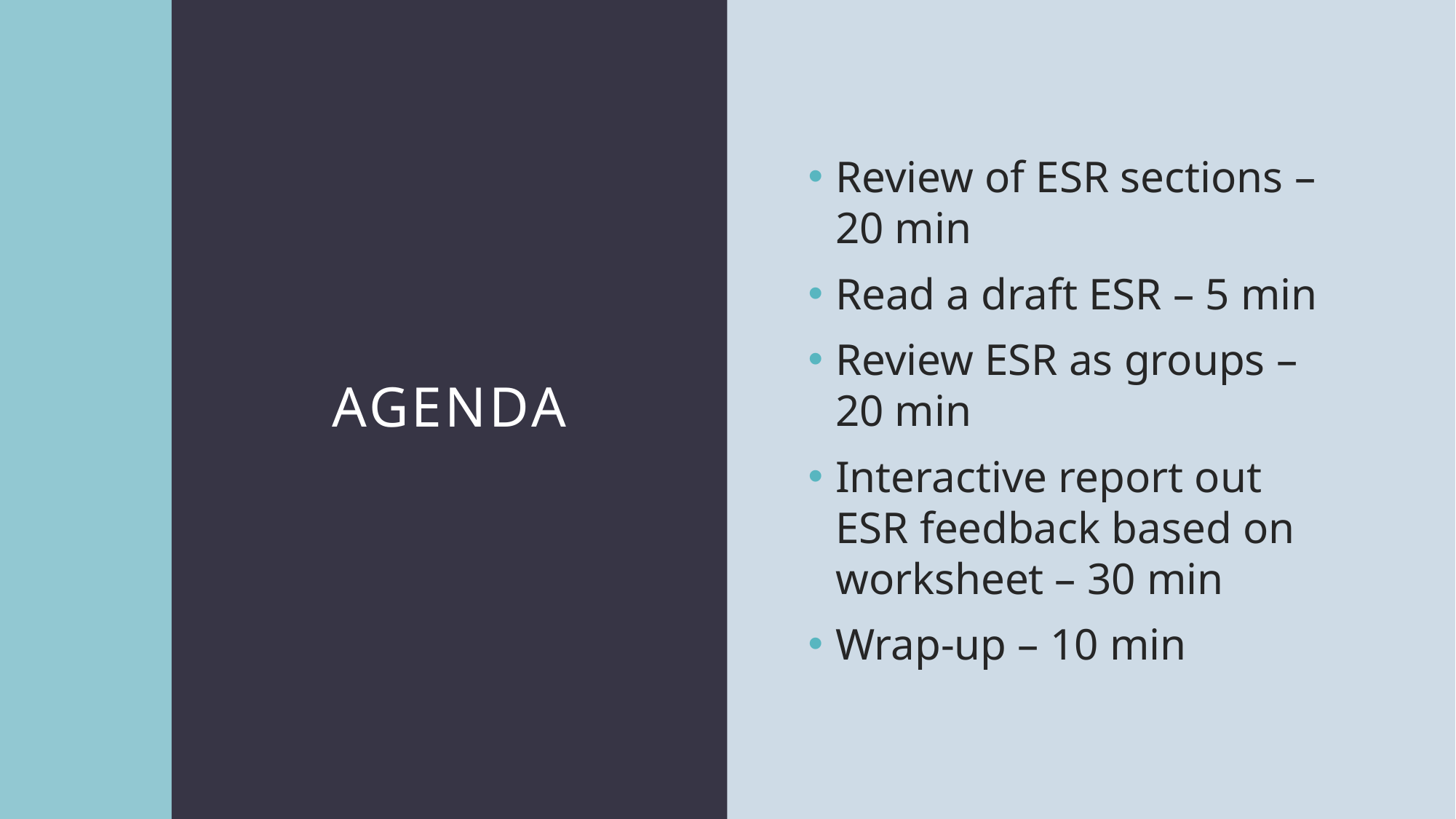

# Agenda
Review of ESR sections – 20 min
Read a draft ESR – 5 min
Review ESR as groups – 20 min
Interactive report out ESR feedback based on worksheet – 30 min
Wrap-up – 10 min

## Slide 4
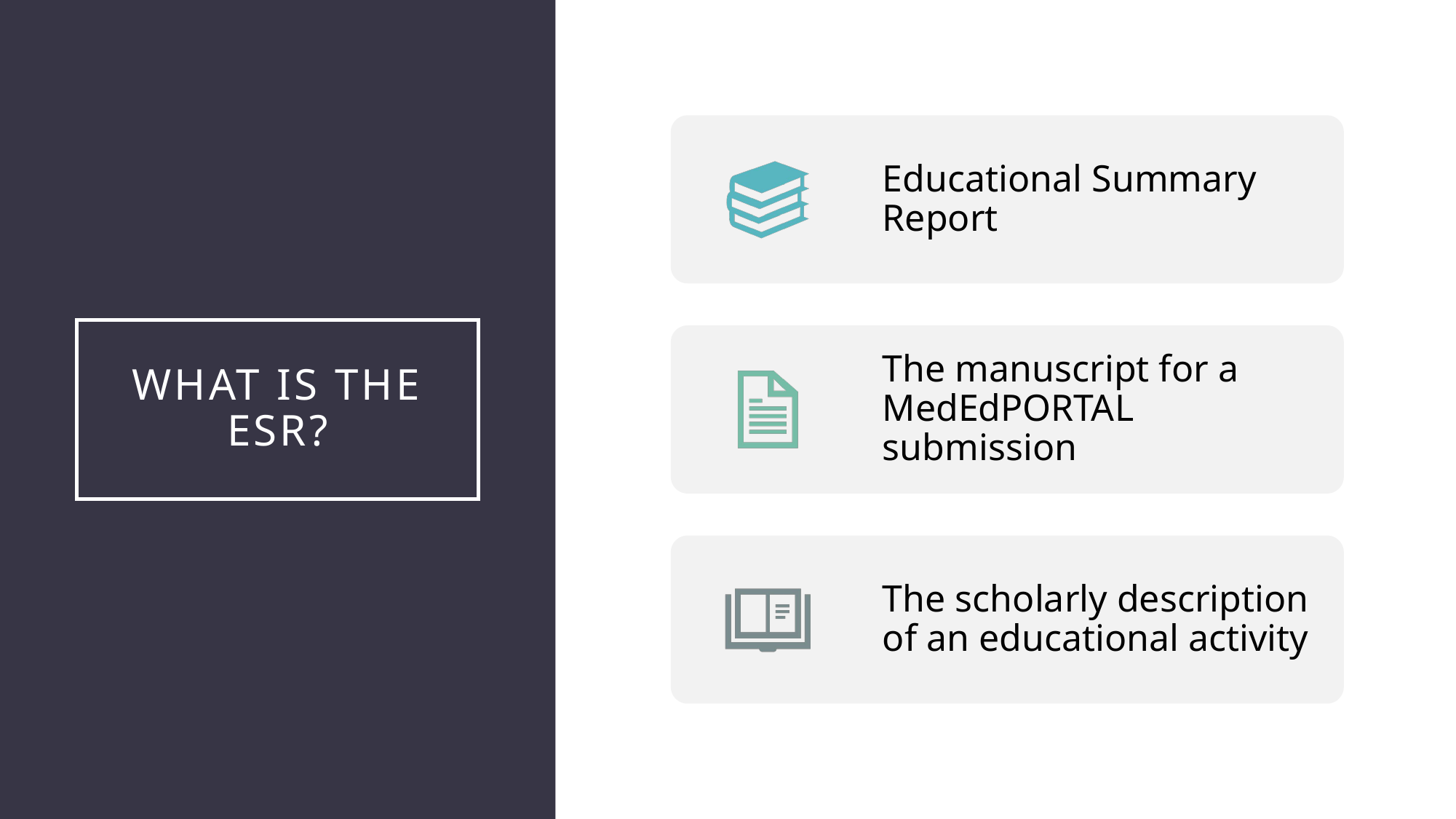

# What is the esr?

## Slide 5
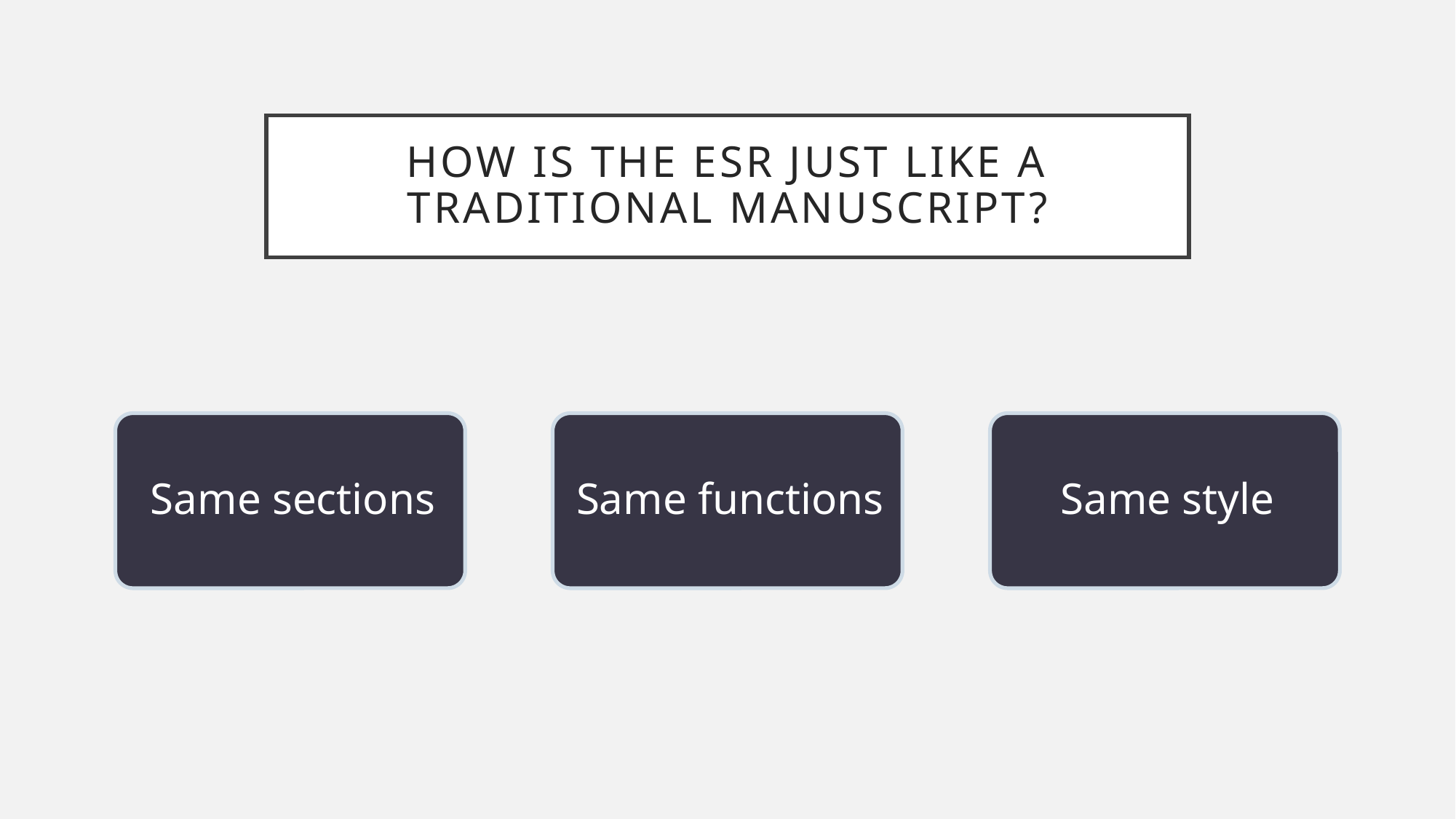

# how is the ESR just like a traditional manuscript?

## Slide 6
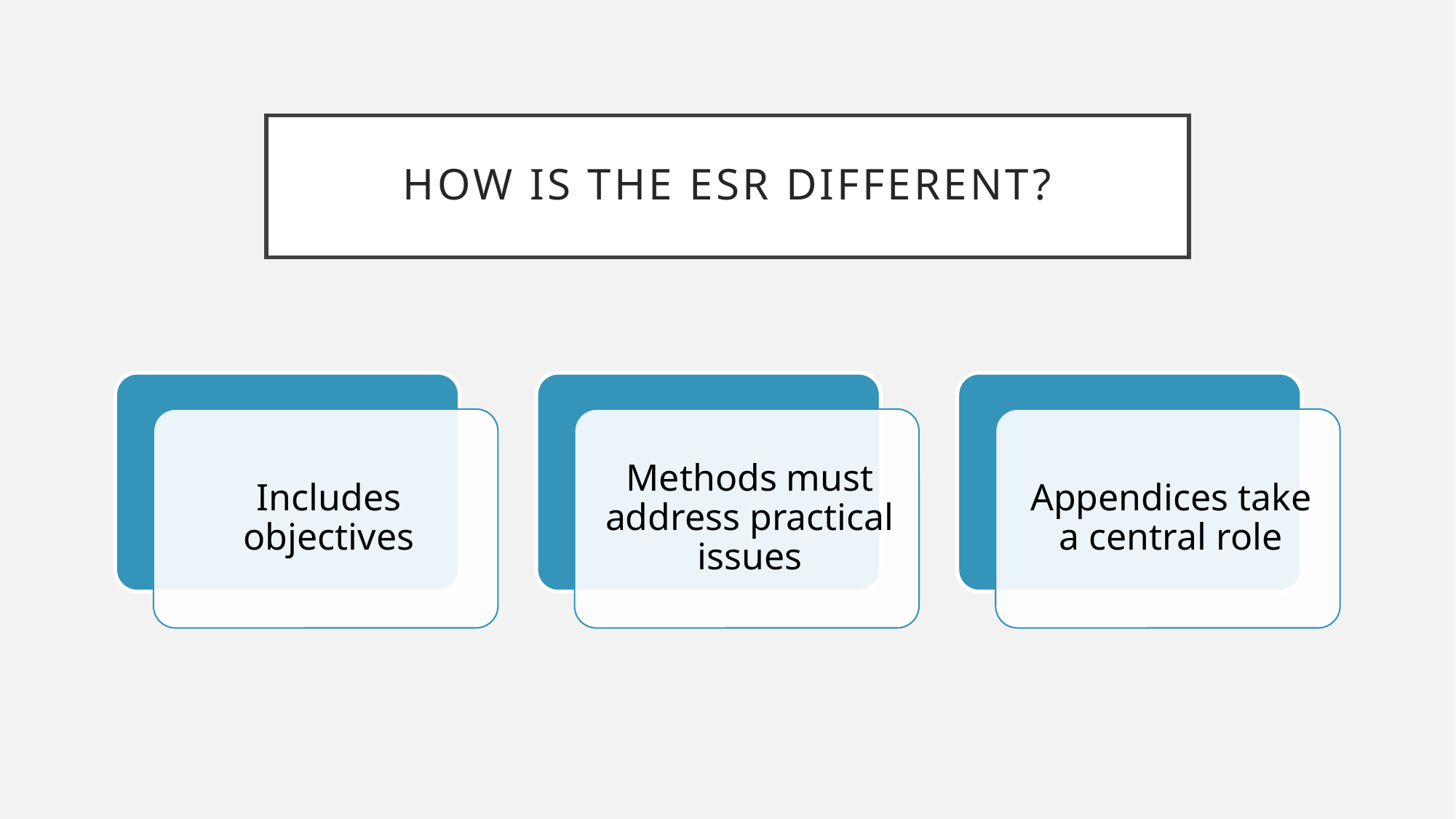

# how is the esr different?

## Slide 7
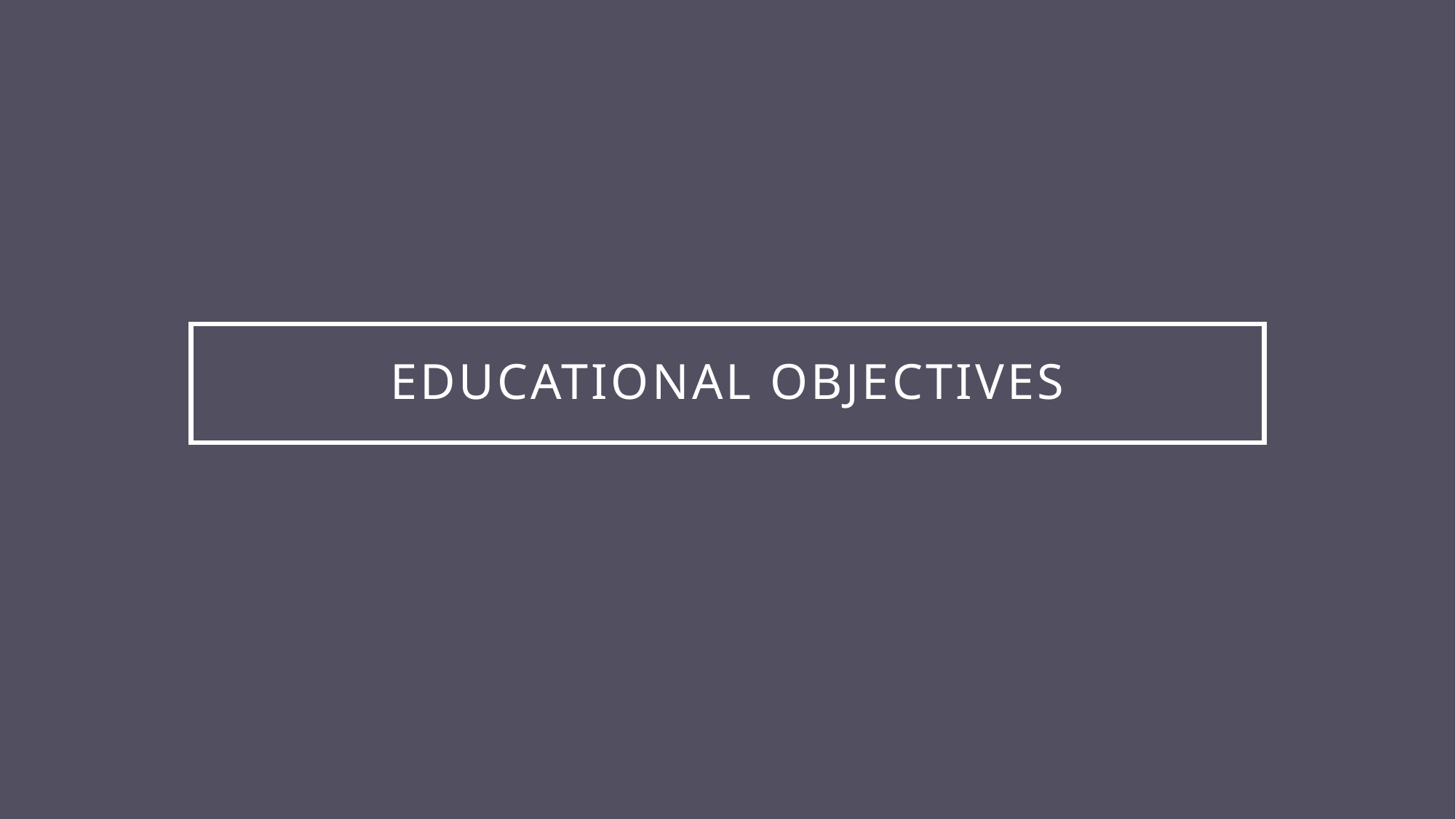

# Educational objectives

## Slide 8
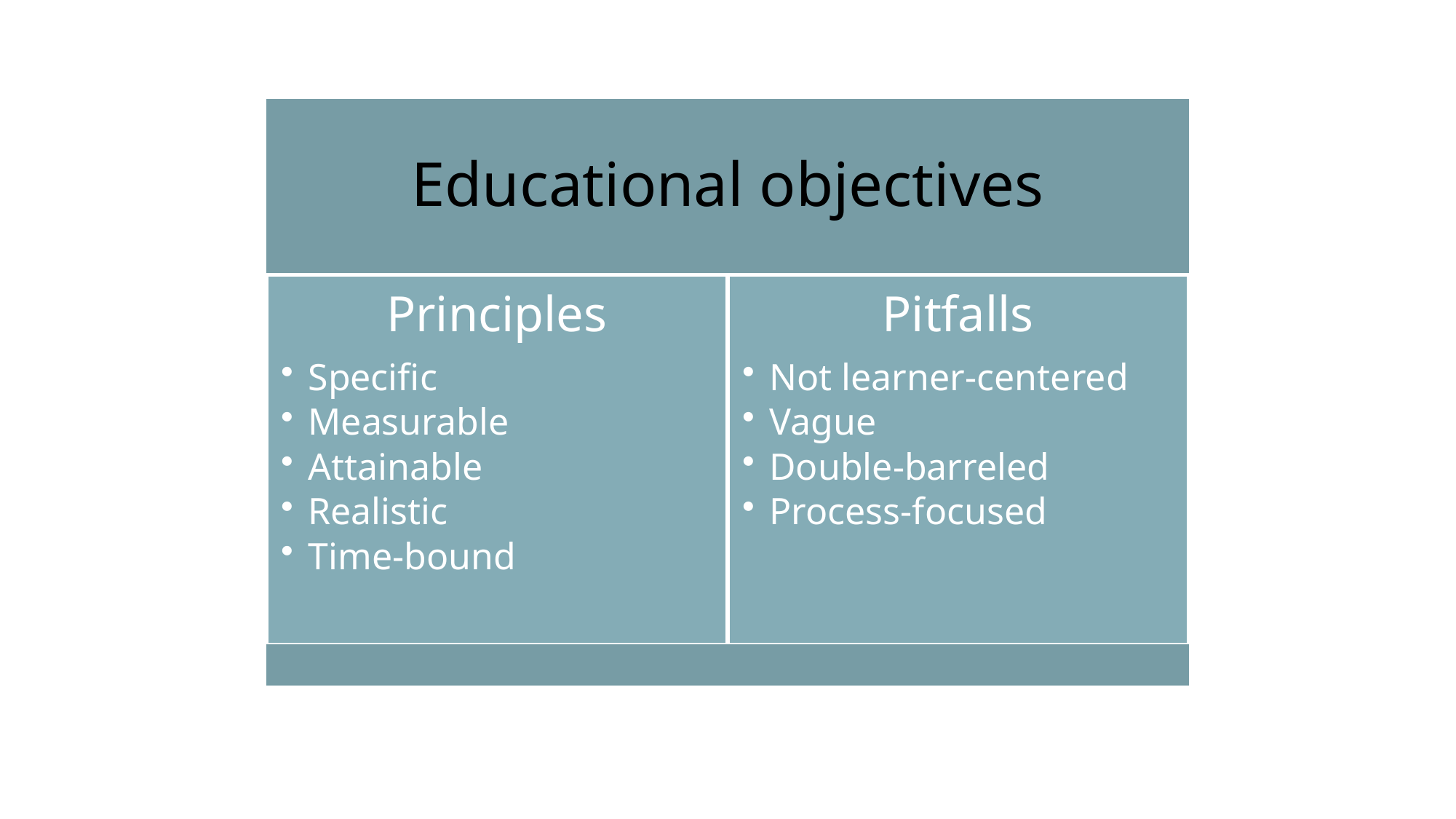

## Slide 9
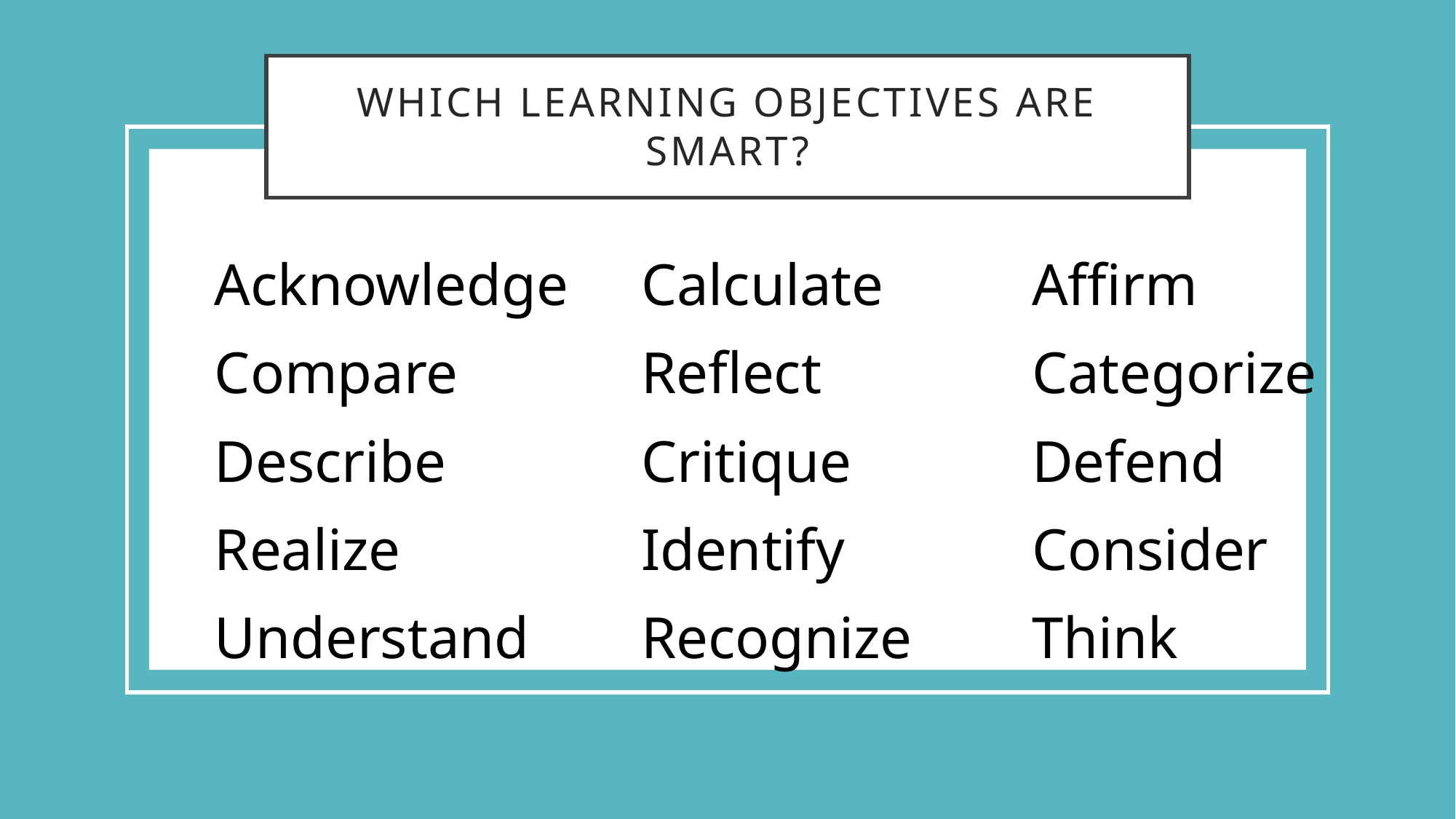

# Which learning objectives are SMART?
Acknowledge
Compare
Describe
Realize
Understand
Calculate
Reflect
Critique
Identify
Recognize
Affirm
Categorize
Defend
Consider
Think

## Slide 10
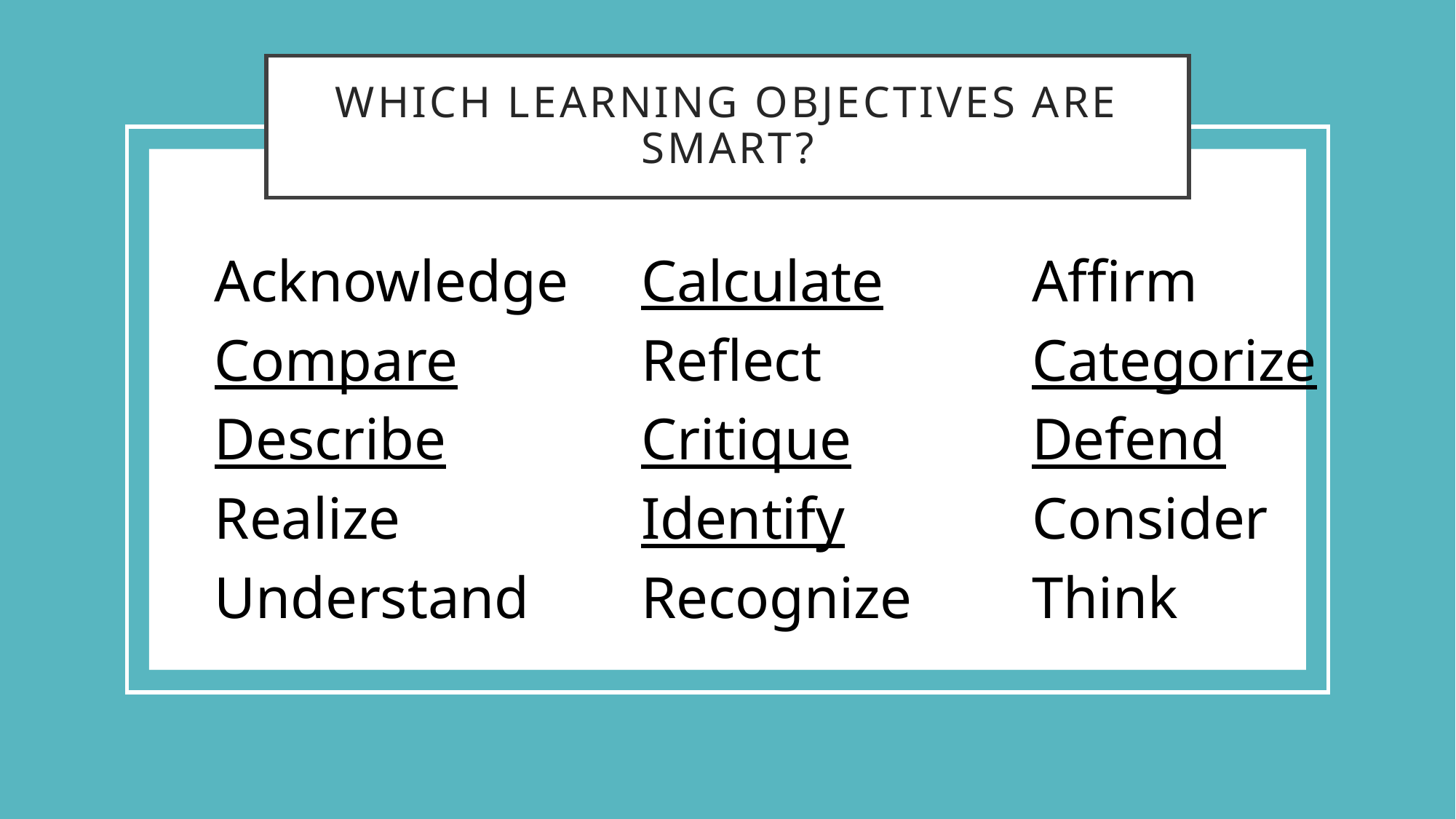

# Which learning objectives are SMART?
Acknowledge
Compare
Describe
Realize
Understand
Calculate
Reflect
Critique
Identify
Recognize
Affirm
Categorize
Defend
Consider
Think

## Slide 11
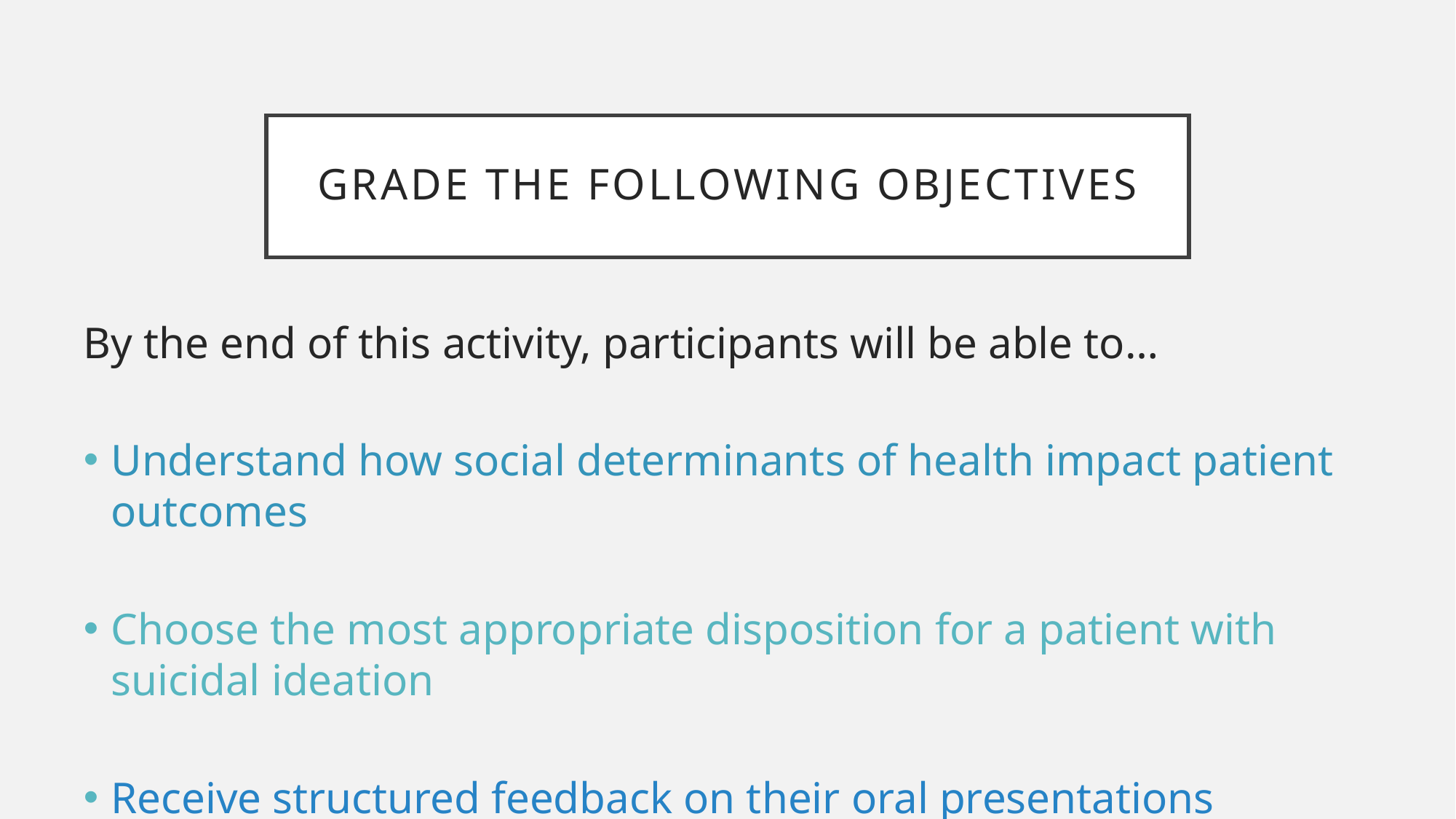

# grade the following objectives
By the end of this activity, participants will be able to…
Understand how social determinants of health impact patient outcomes
Choose the most appropriate disposition for a patient with suicidal ideation
Receive structured feedback on their oral presentations

## Slide 12
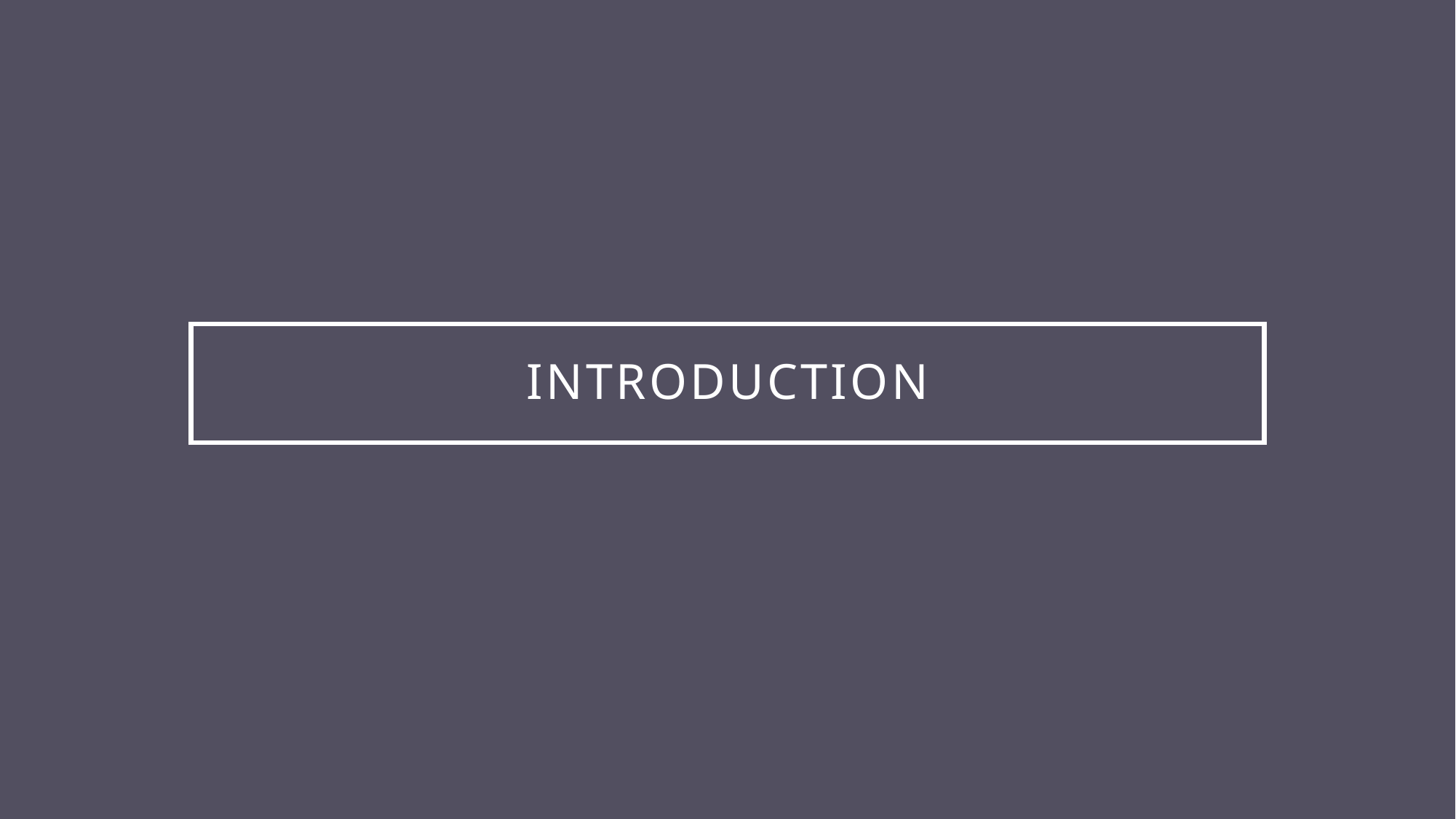

# introduction

## Slide 13
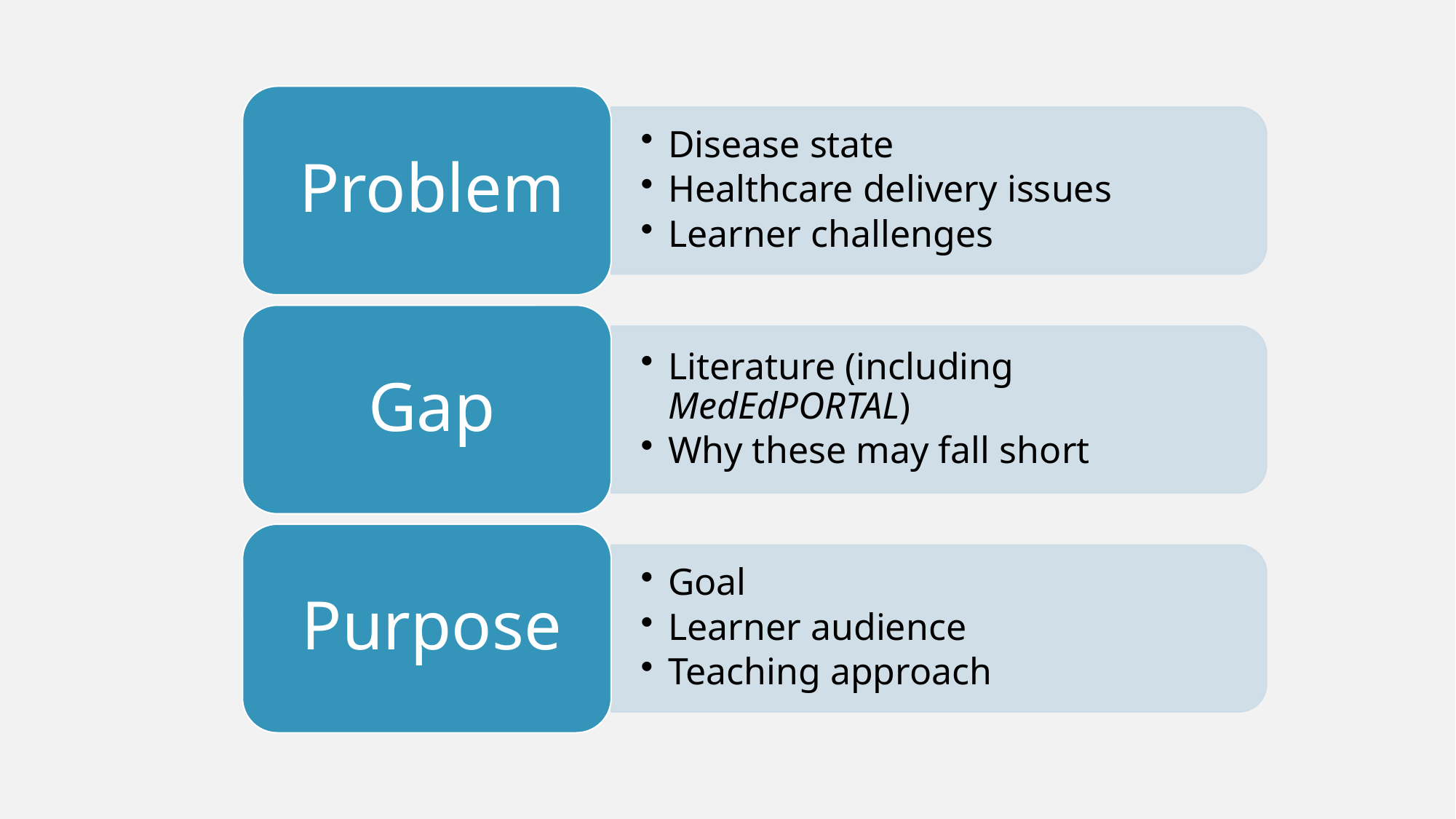

## Slide 14
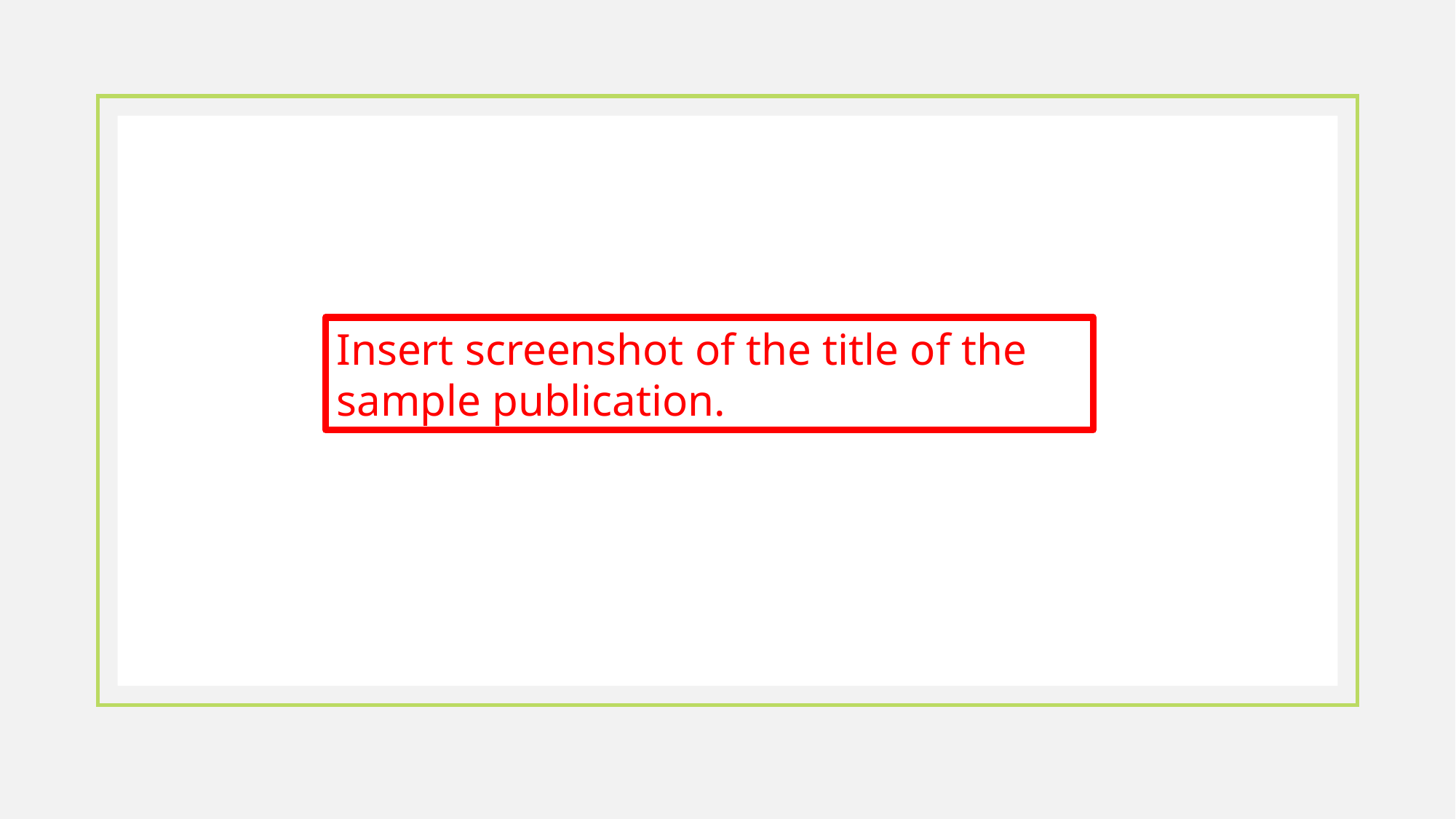

Insert screenshot of the title of the sample publication.

## Slide 15
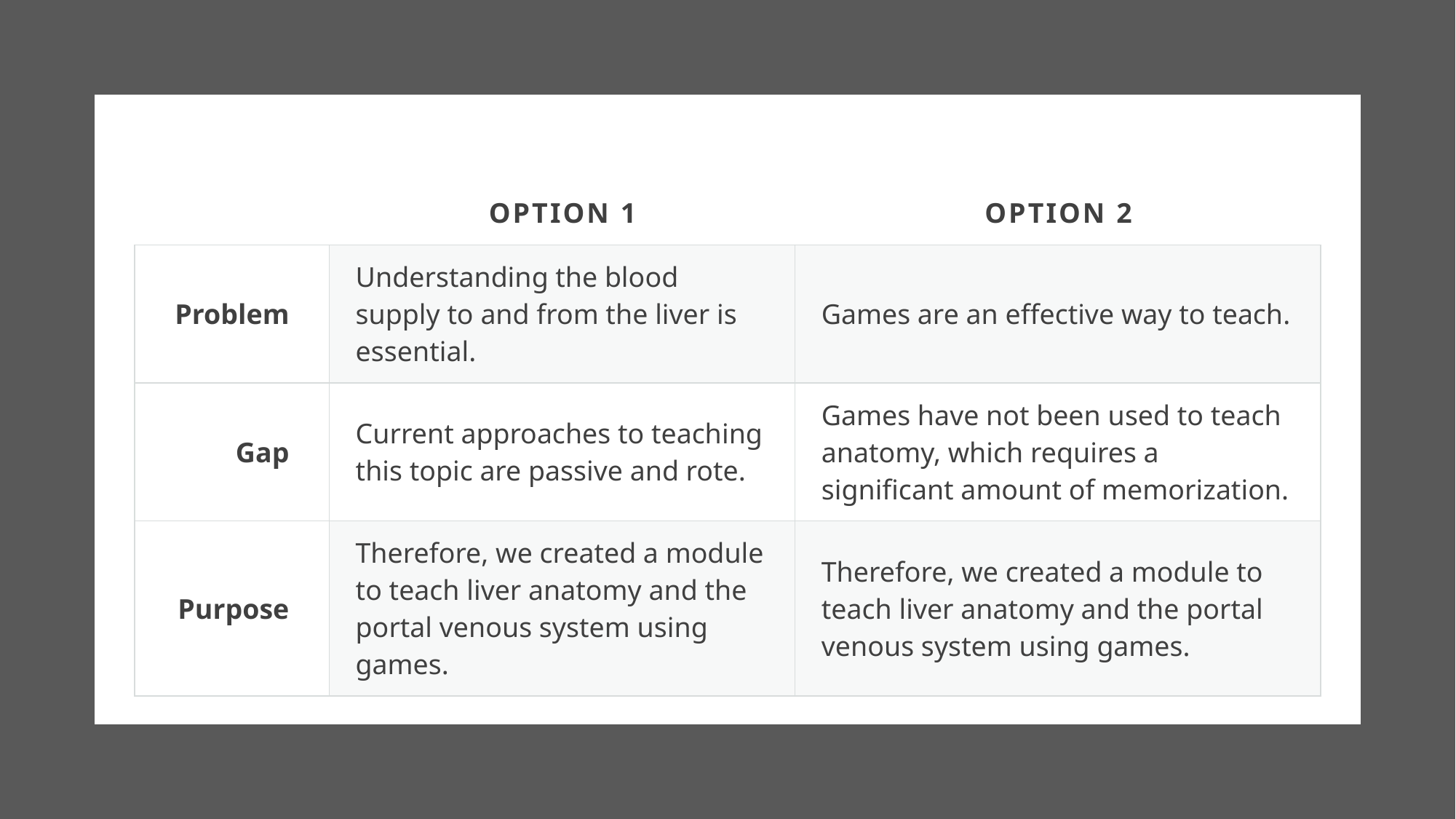

| | Option 1 | Option 2 |
| --- | --- | --- |
| Problem | Understanding the blood supply to and from the liver is essential. | Games are an effective way to teach. |
| Gap | Current approaches to teaching this topic are passive and rote. | Games have not been used to teach anatomy, which requires a significant amount of memorization. |
| Purpose | Therefore, we created a module to teach liver anatomy and the portal venous system using games. | Therefore, we created a module to teach liver anatomy and the portal venous system using games. |

## Slide 16
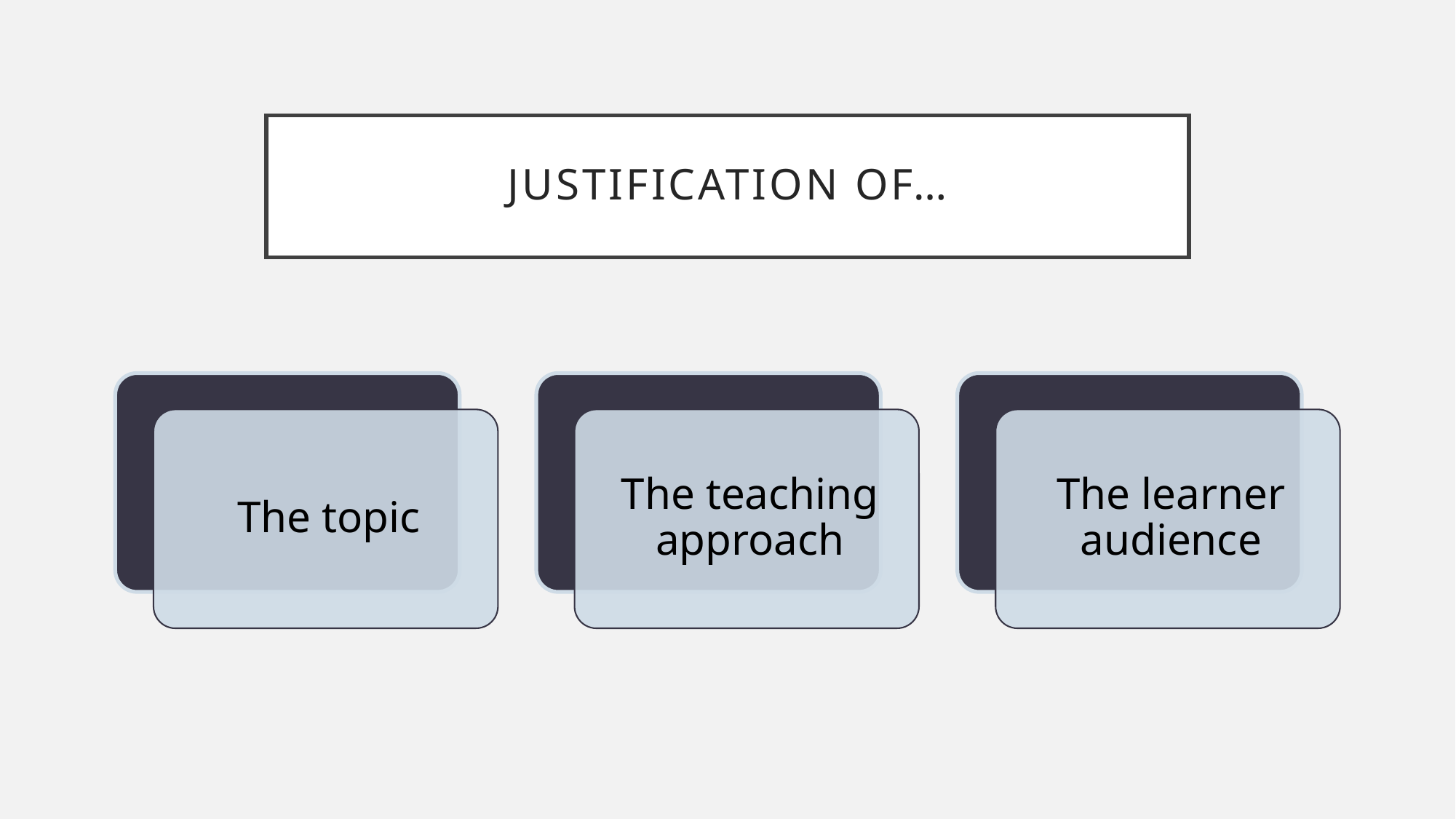

# justification of…

## Slide 17
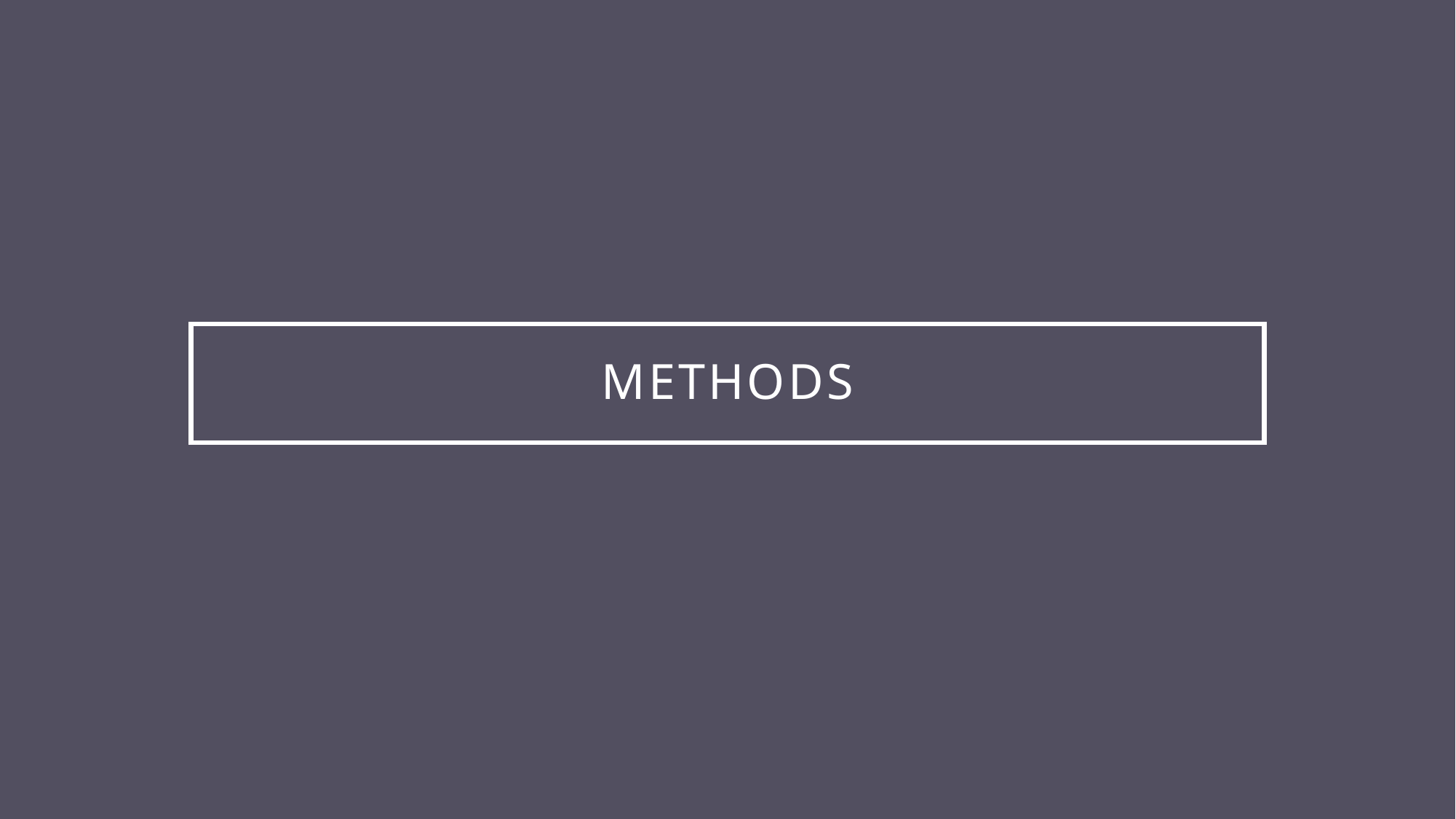

# methods

## Slide 18
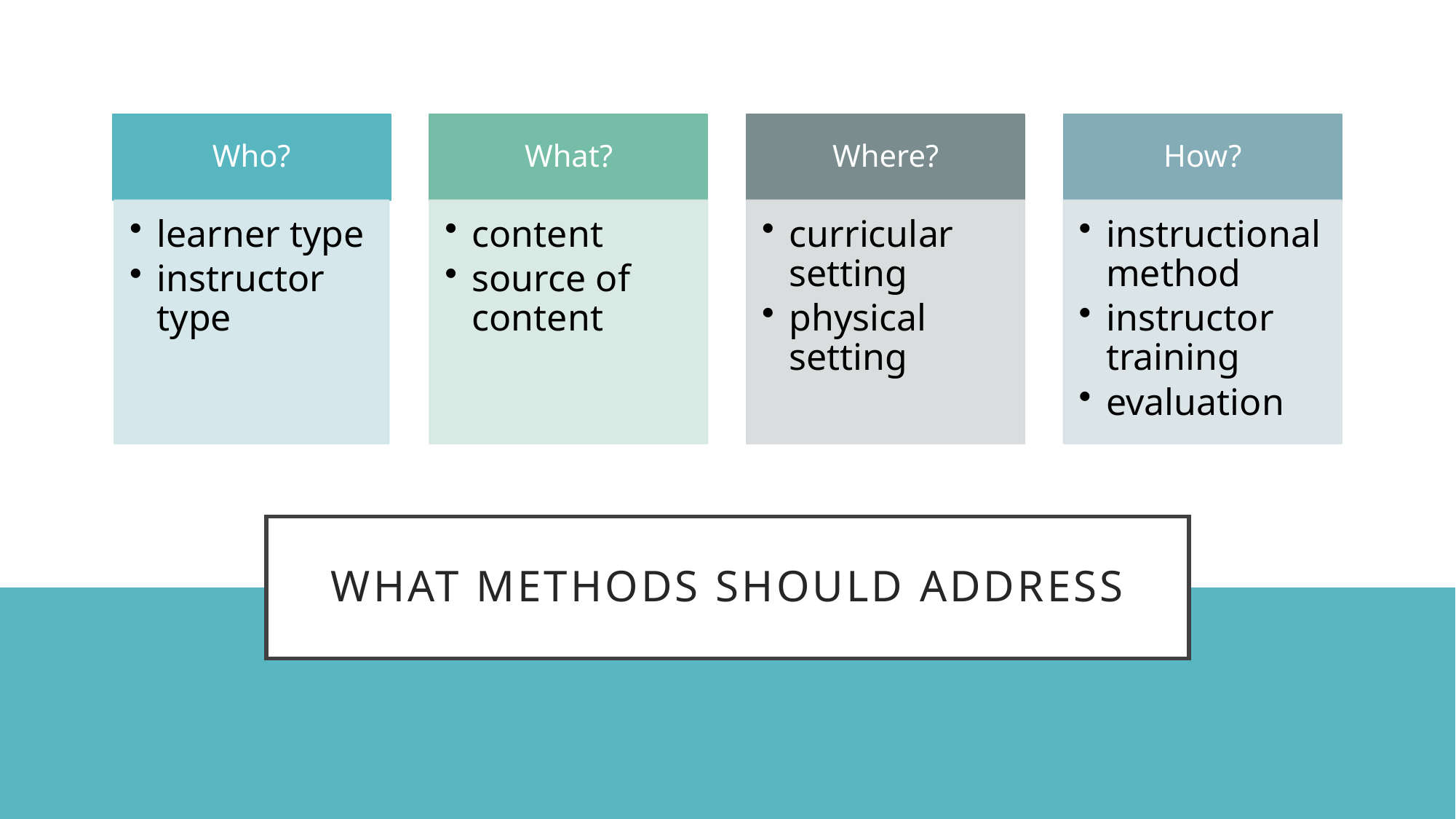

# what methods should address

## Slide 19
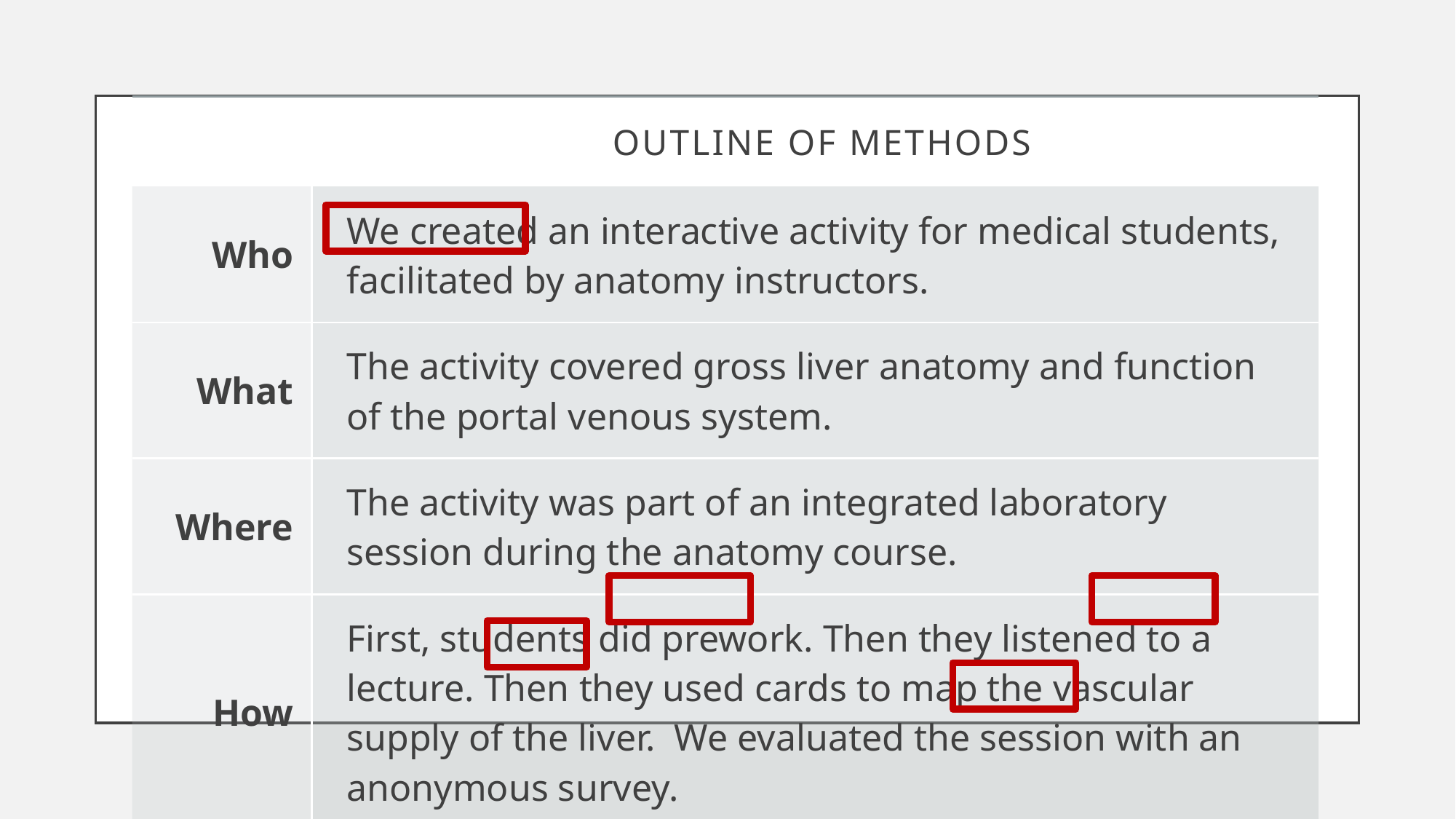

| | Outline of methods |
| --- | --- |
| Who | We created an interactive activity for medical students, facilitated by anatomy instructors. |
| What | The activity covered gross liver anatomy and function of the portal venous system. |
| Where | The activity was part of an integrated laboratory session during the anatomy course. |
| How | First, students did prework. Then they listened to a lecture. Then they used cards to map the vascular supply of the liver. We evaluated the session with an anonymous survey. |

## Slide 20
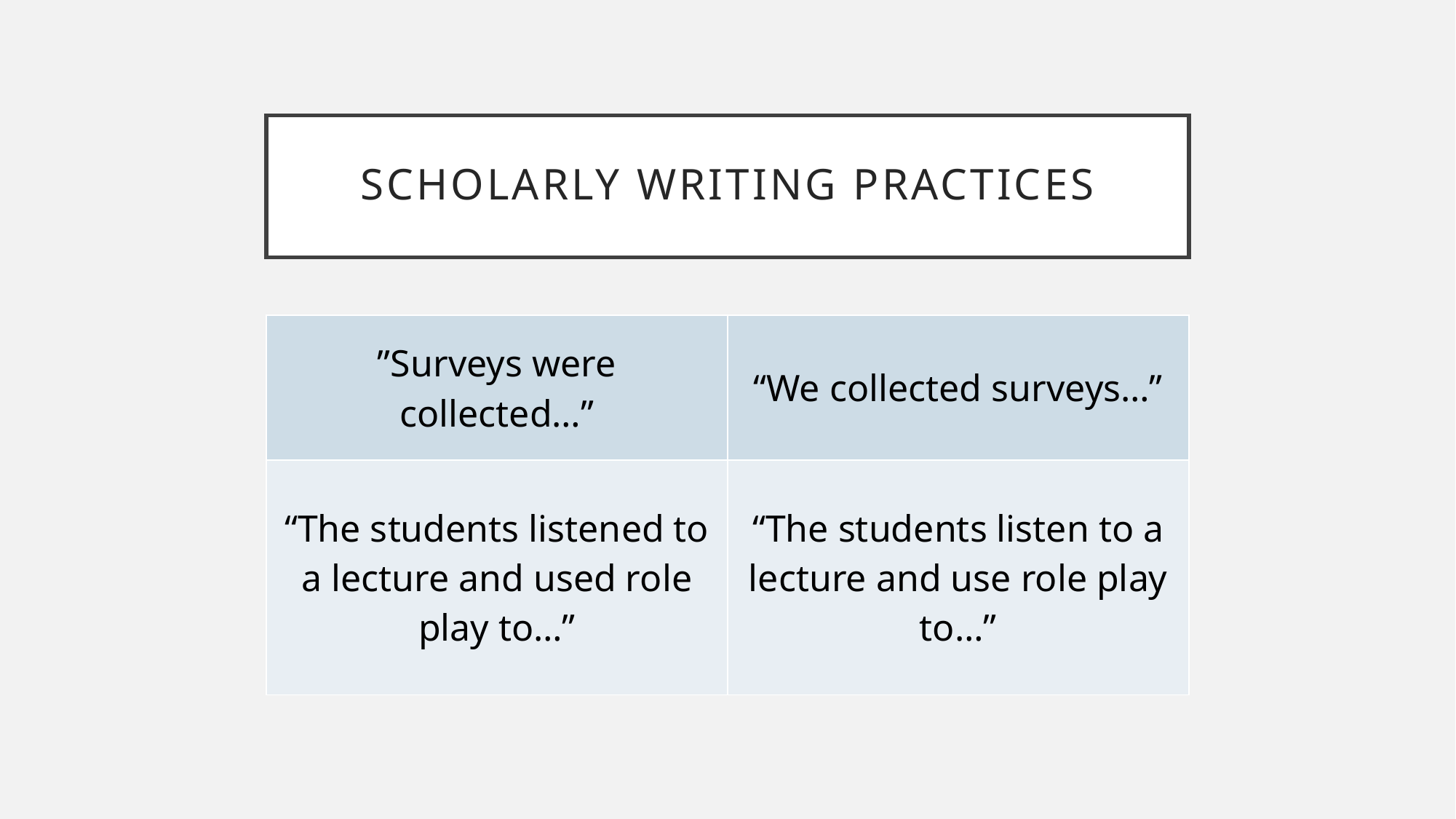

# Scholarly writing practices
| ”Surveys were collected…” | “We collected surveys…” |
| --- | --- |
| “The students listened to a lecture and used role play to…” | “The students listen to a lecture and use role play to…” |

## Slide 21
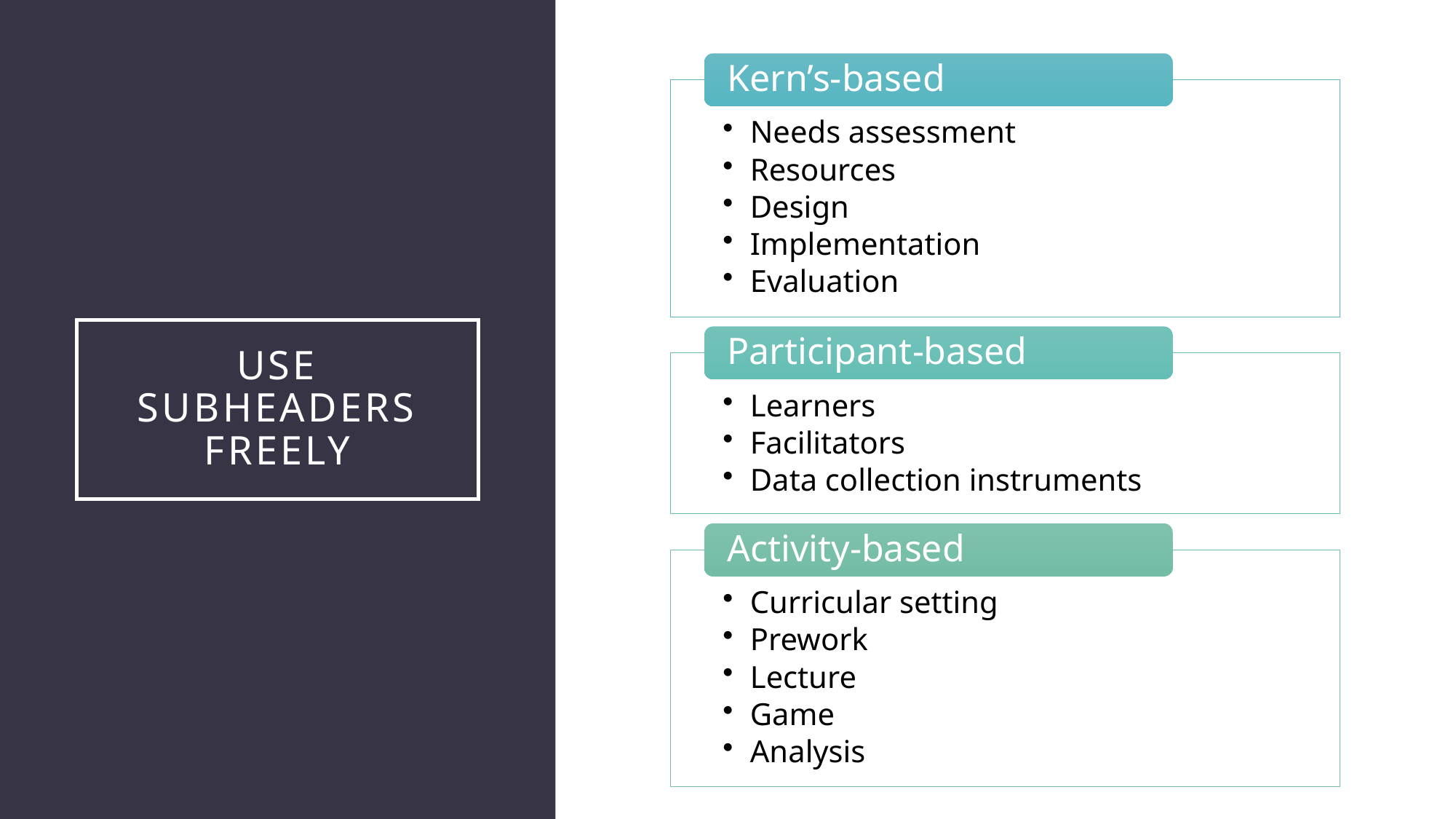

# Use subheaders freely

## Slide 22
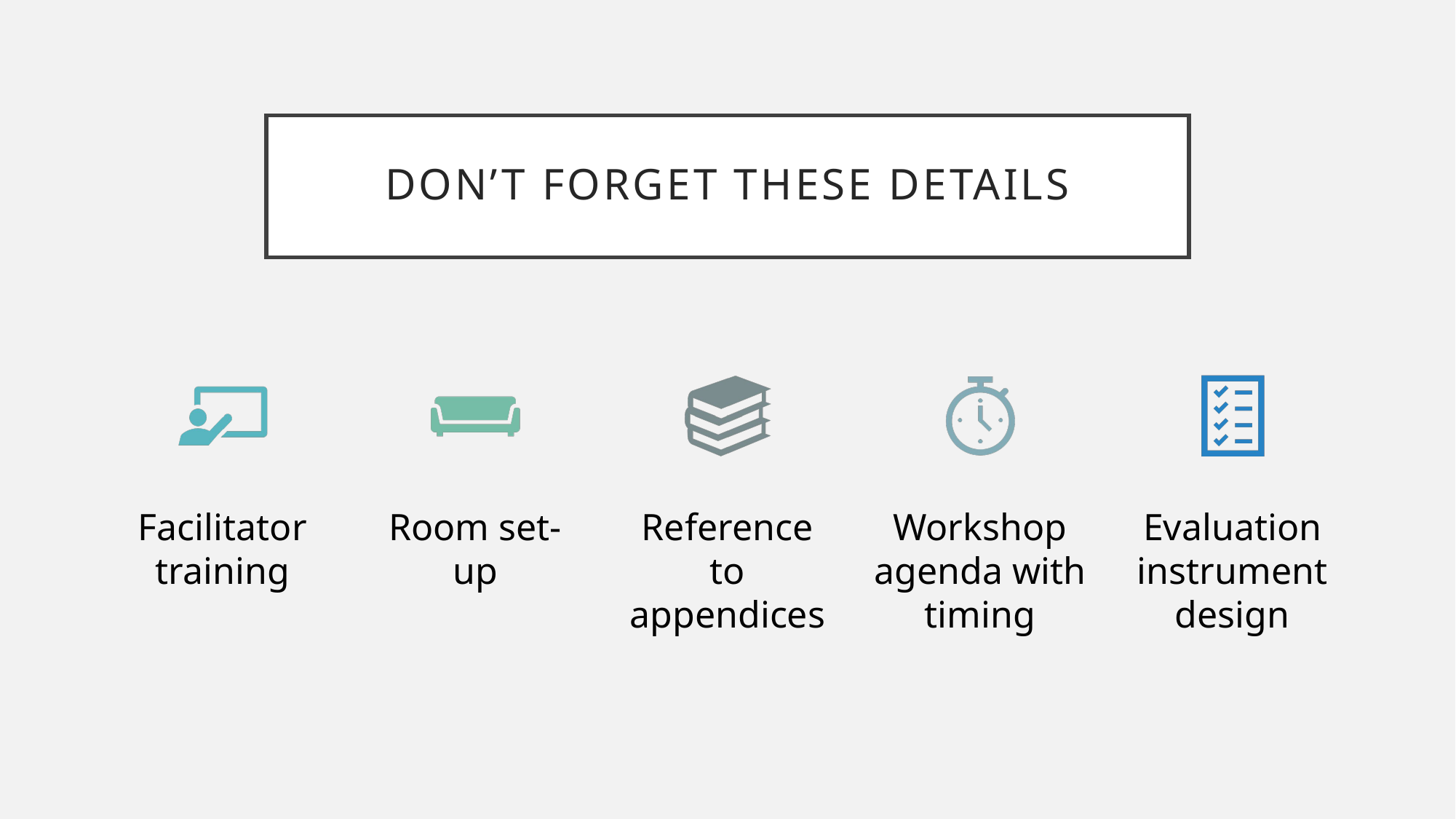

# don’t forget these details

## Slide 23
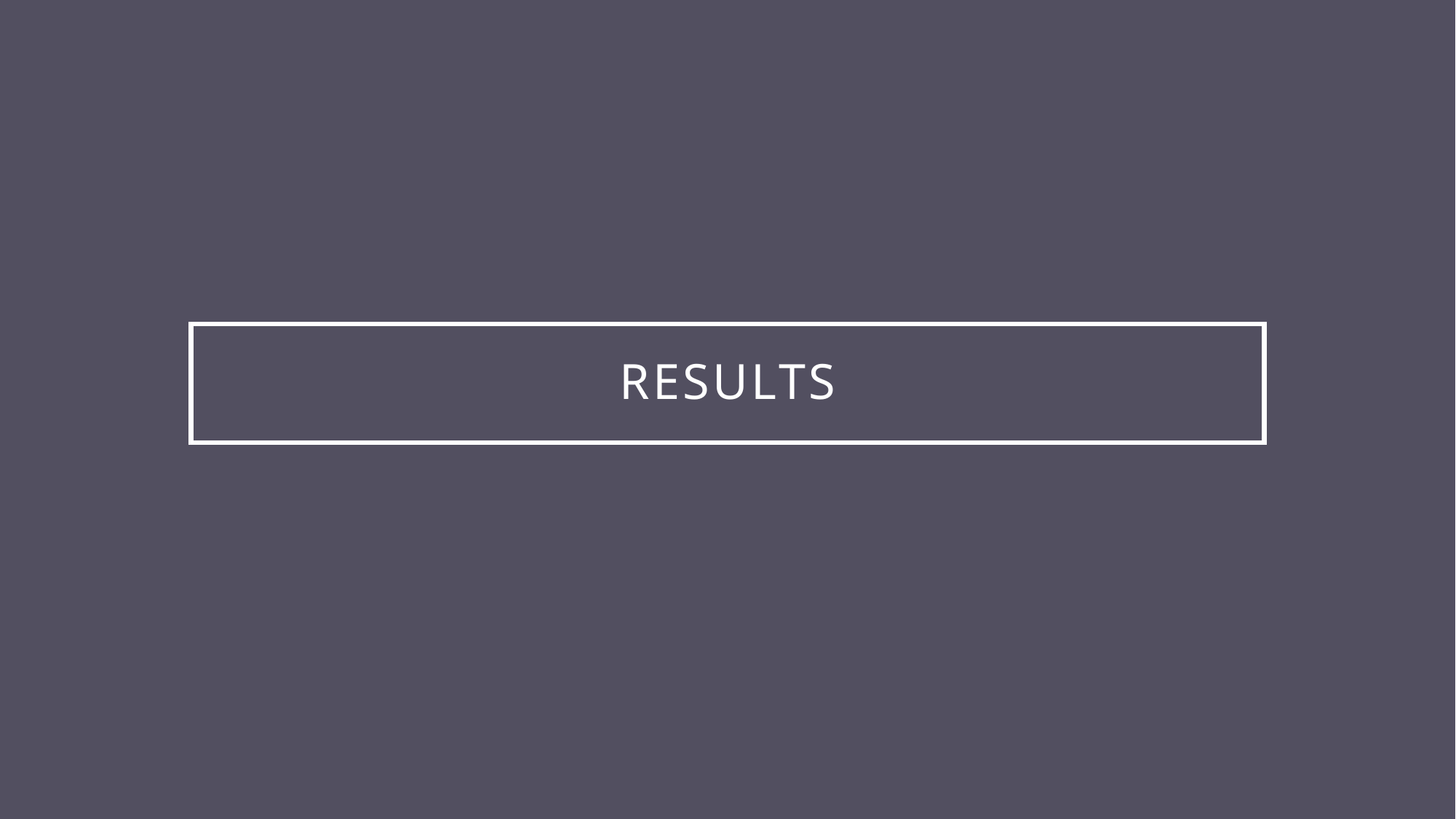

# results

## Slide 24
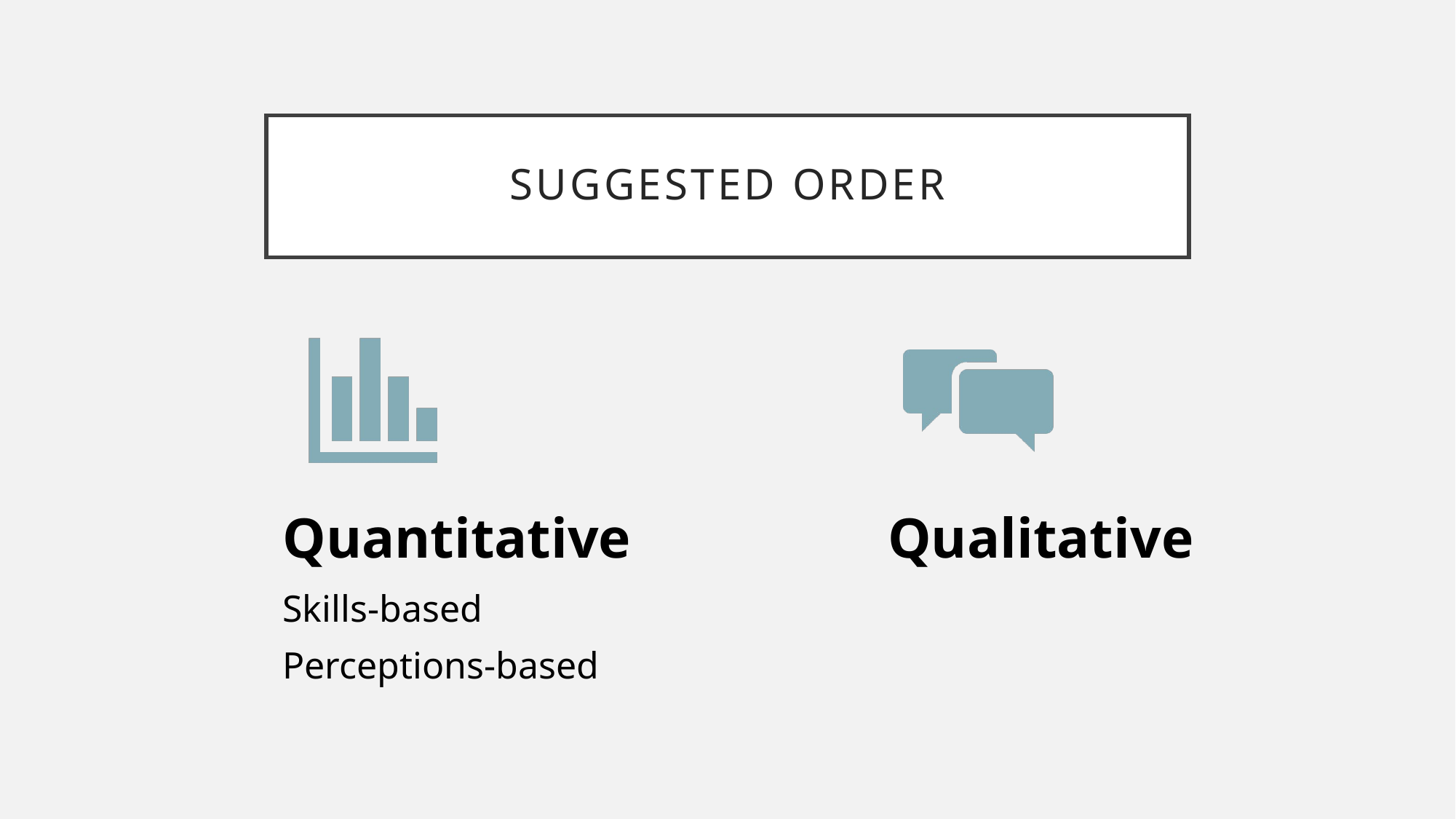

# suggested order

## Slide 25
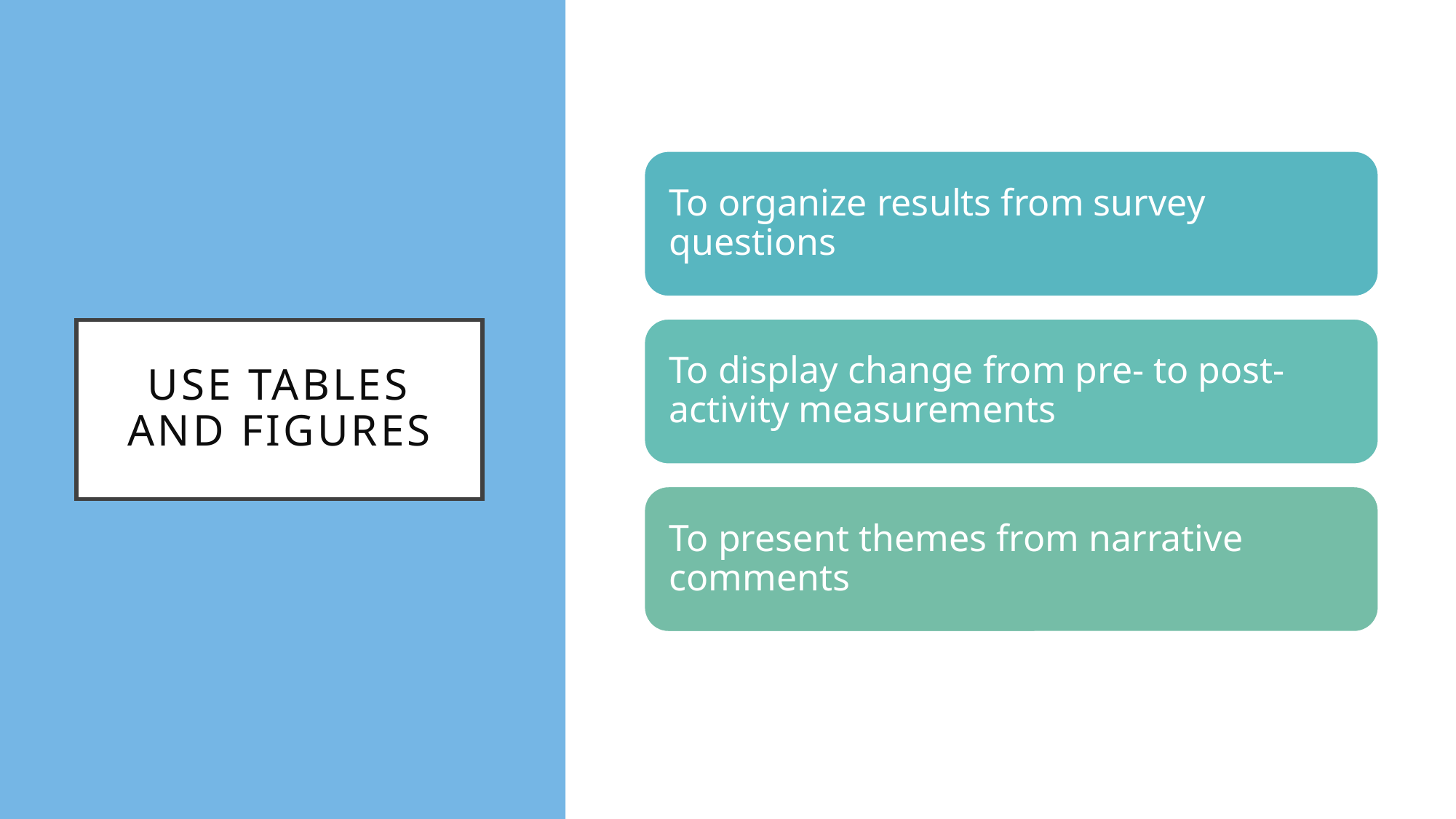

# Use tables and figures

## Slide 26
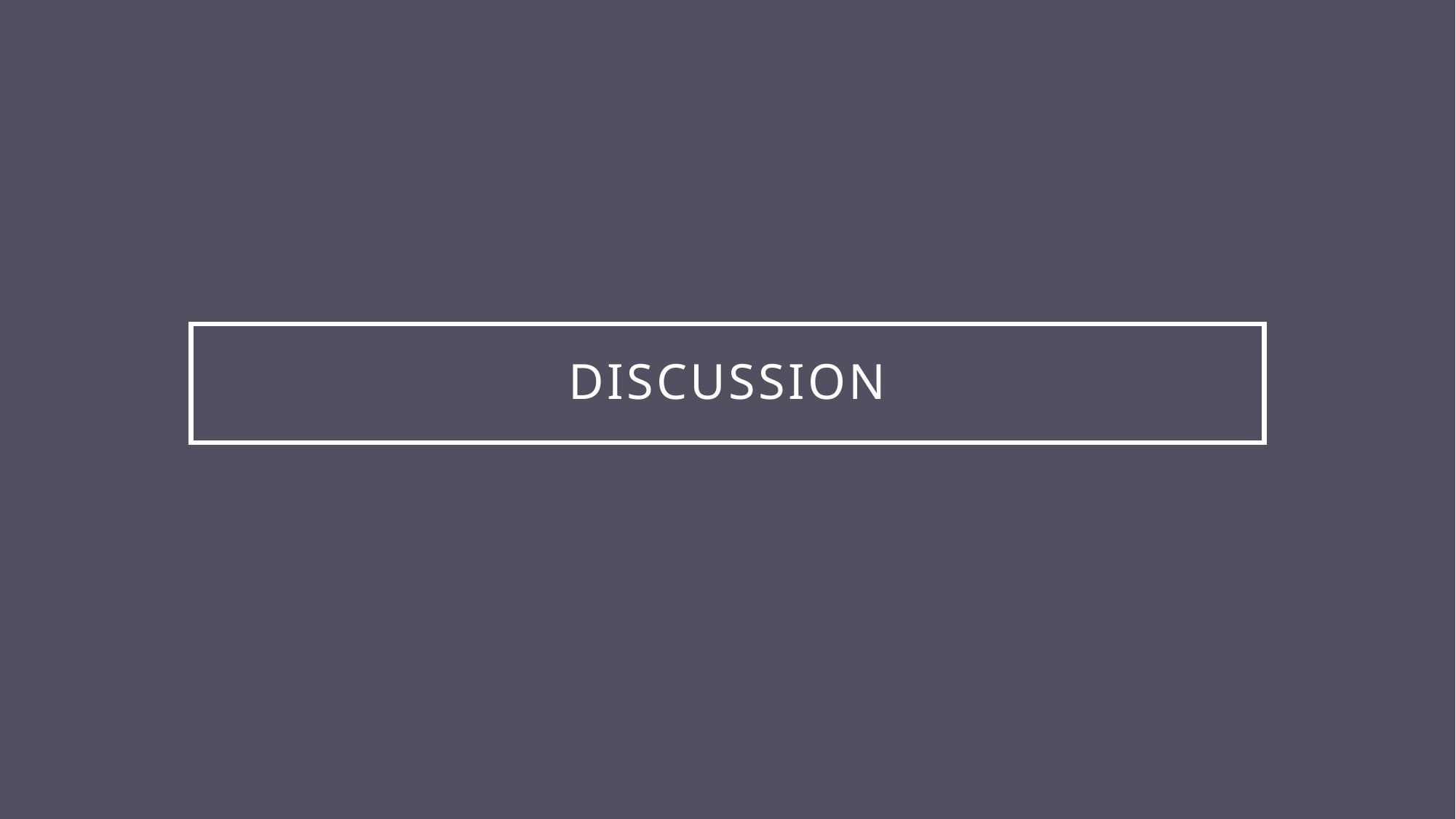

# discussion

## Slide 27
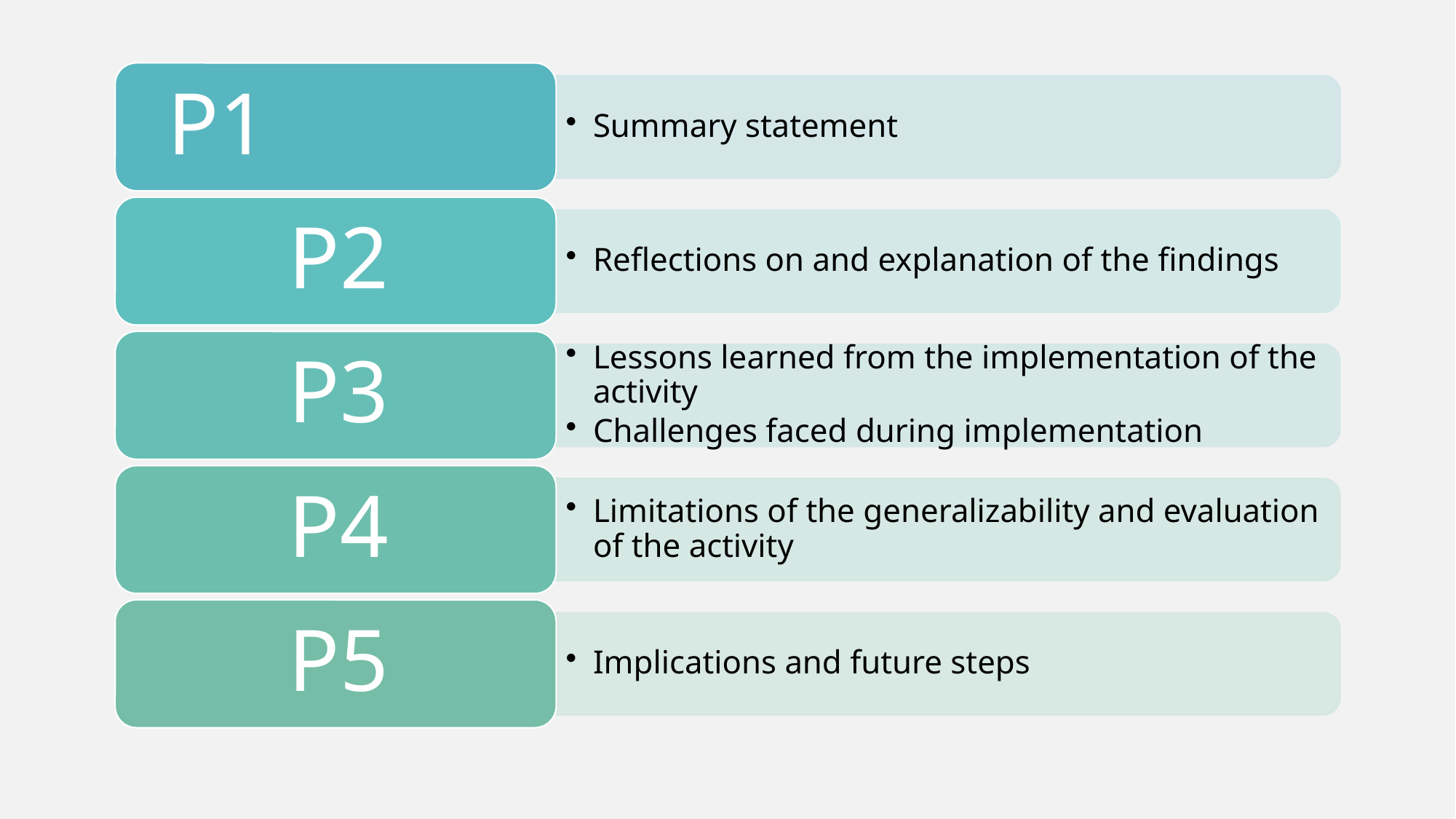

## Slide 28
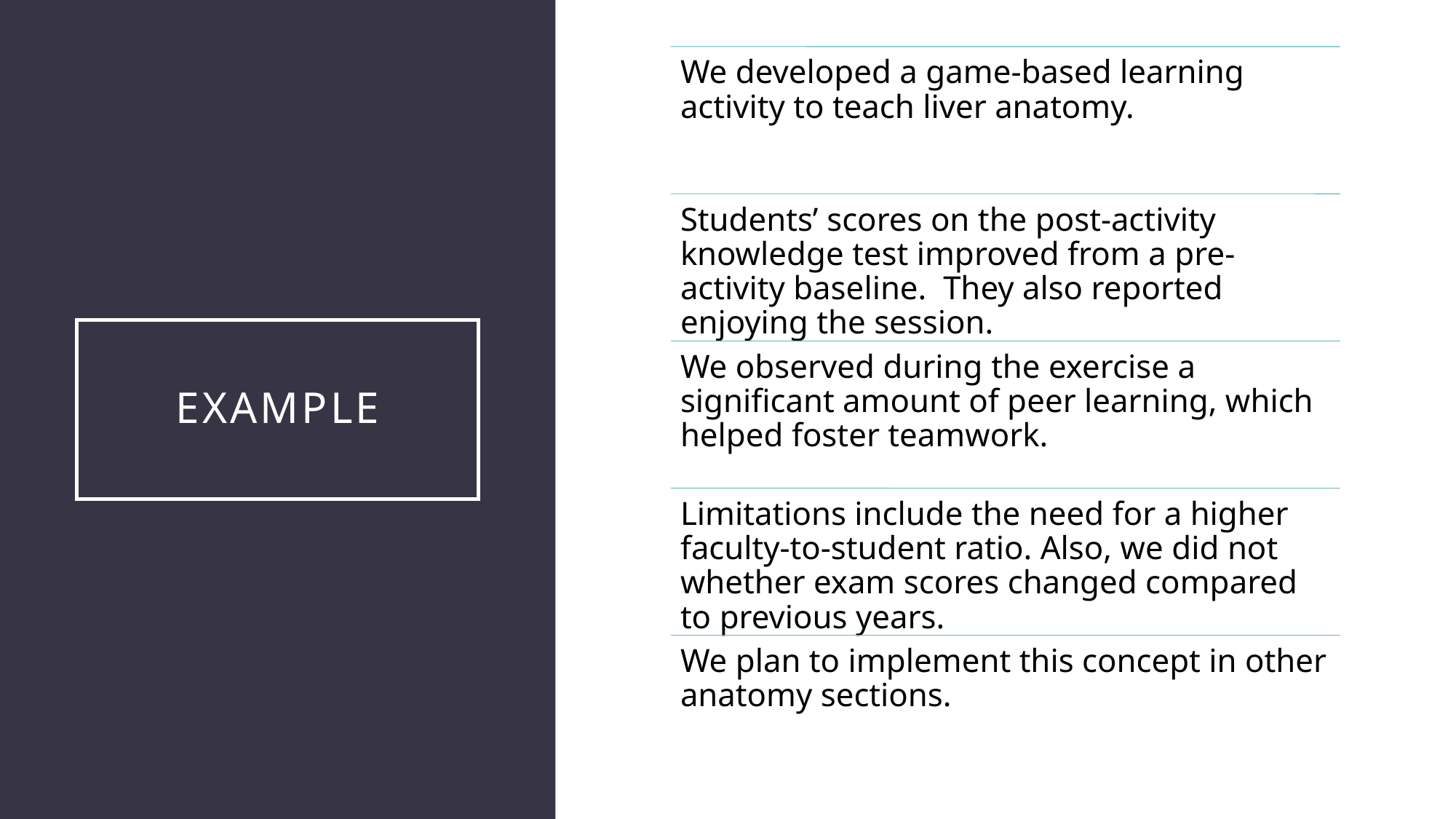

# Example

## Slide 29
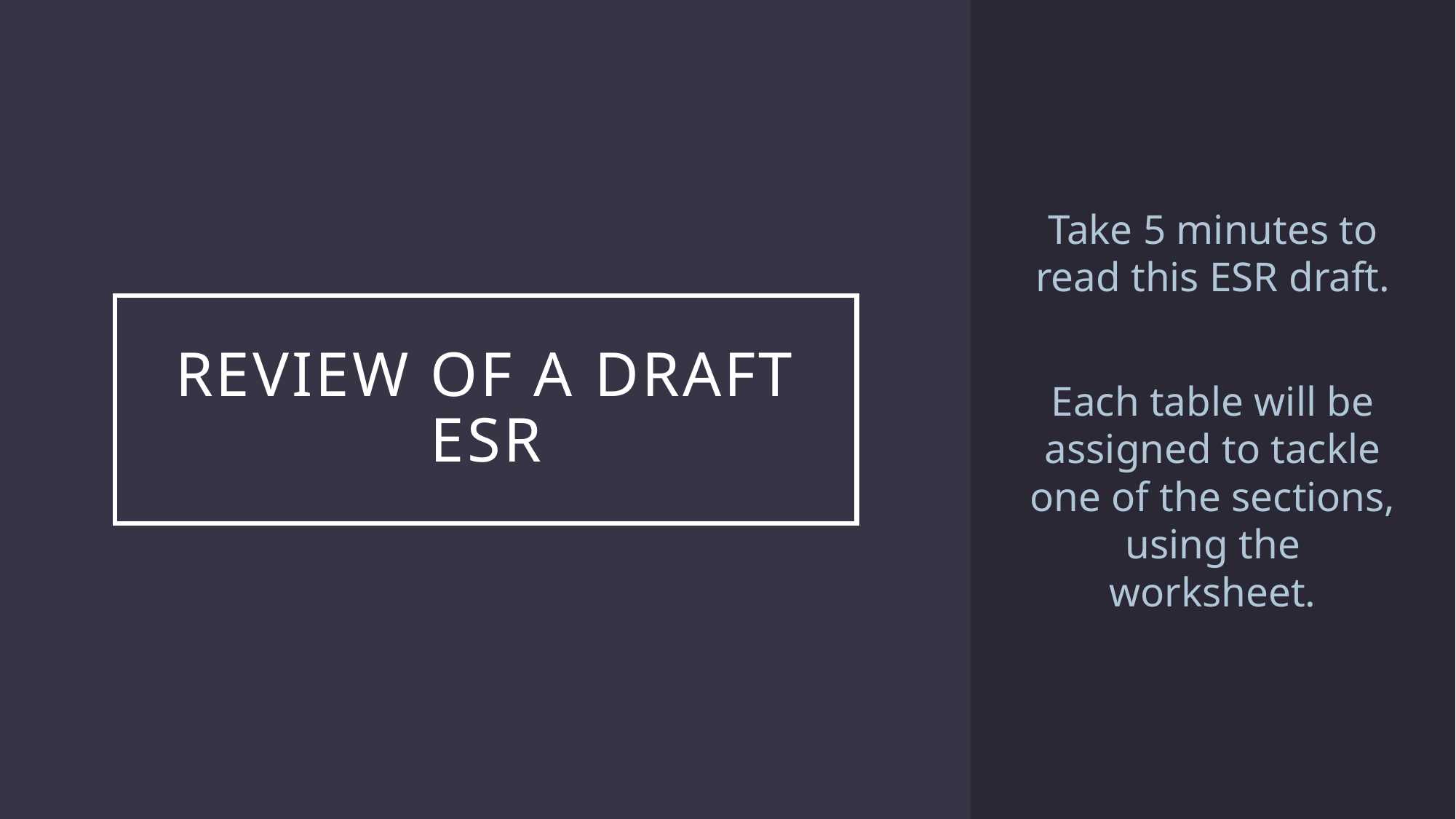

Take 5 minutes to read this ESR draft.
Each table will be assigned to tackle one of the sections, using the worksheet.
# Review of a draft ESR

## Slide 30
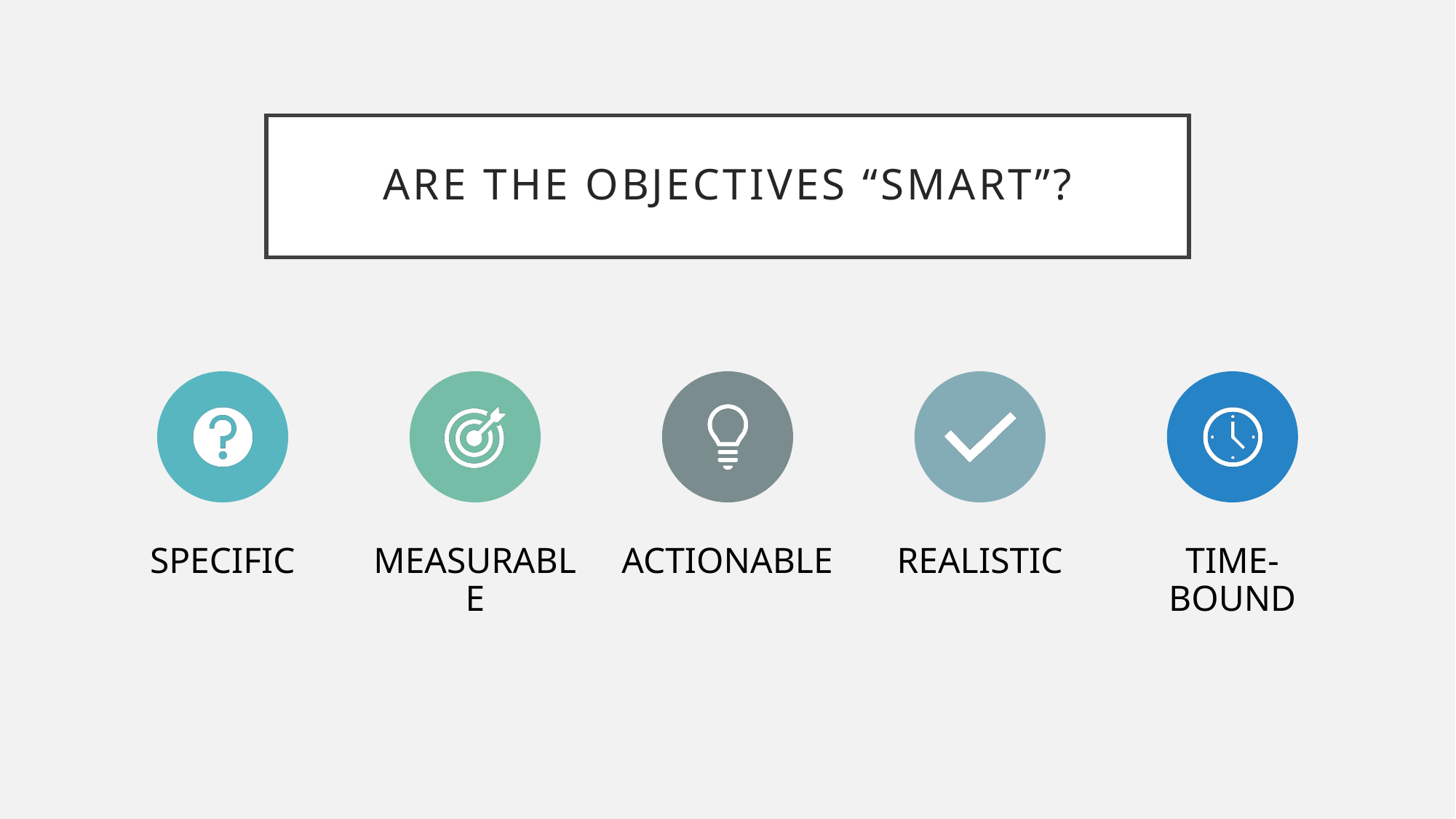

# Are the objectives “smart”?

## Slide 31
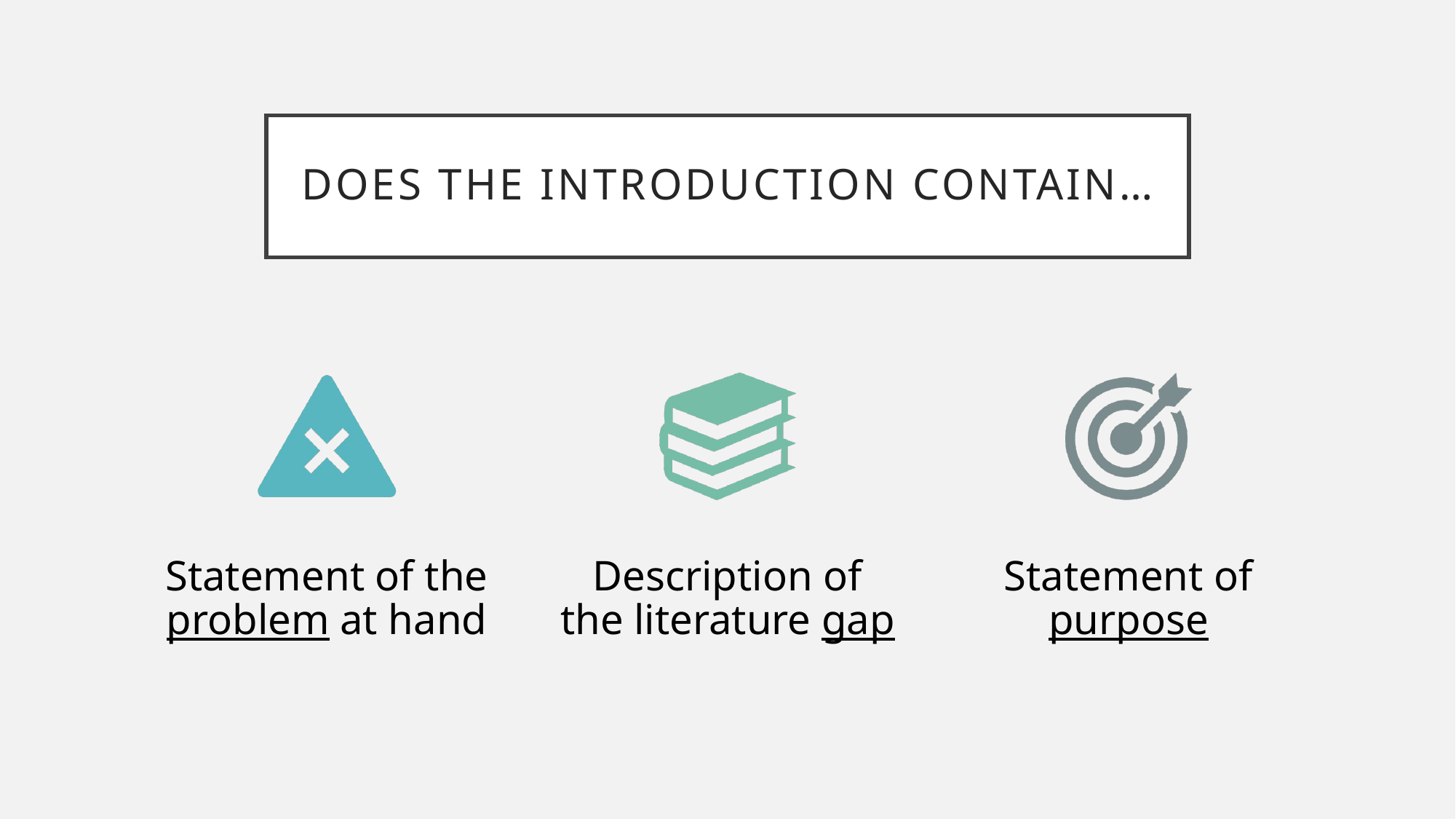

# Does the introduction contain…

## Slide 32
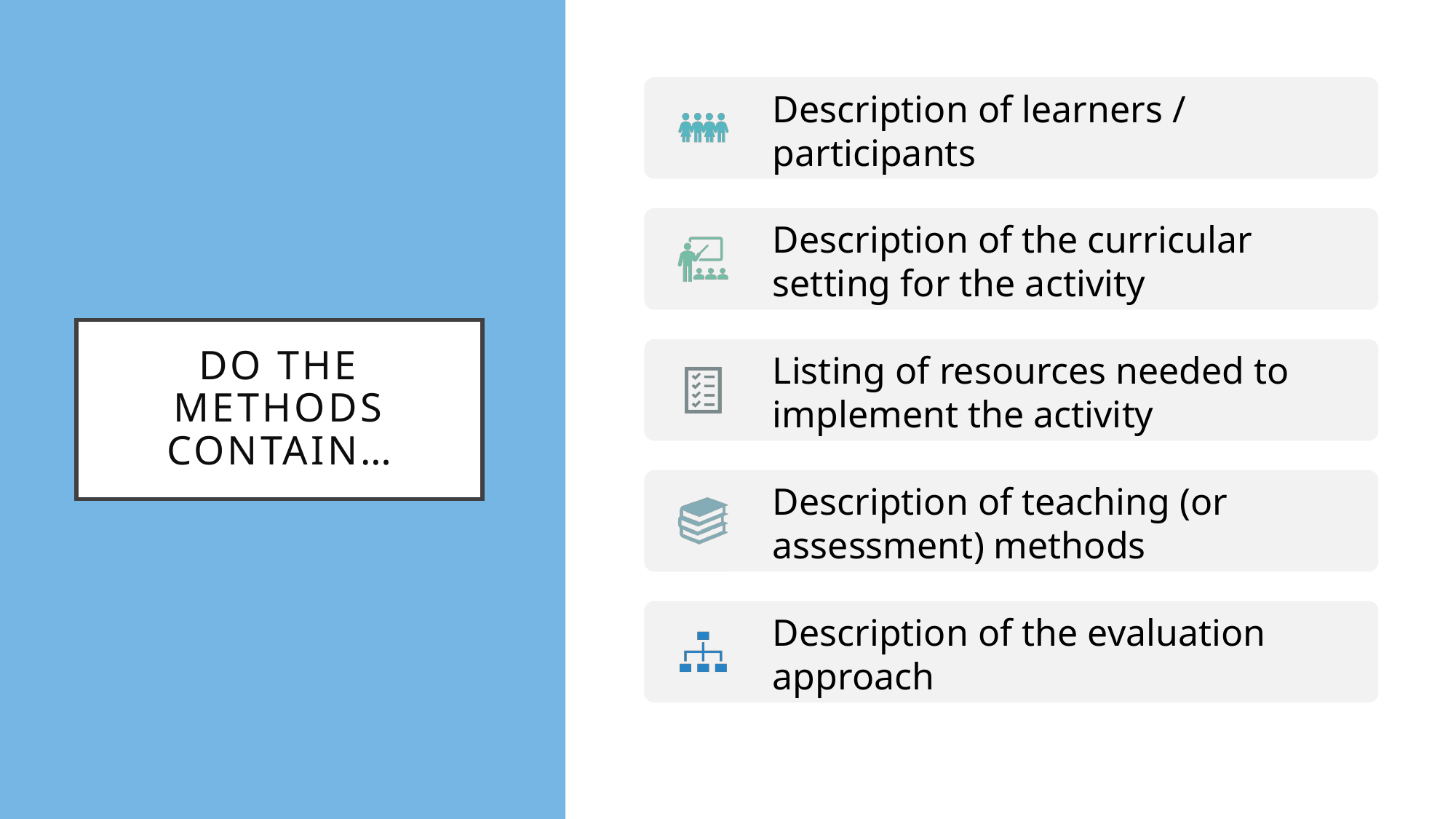

# Do the Methods contain…

## Slide 33
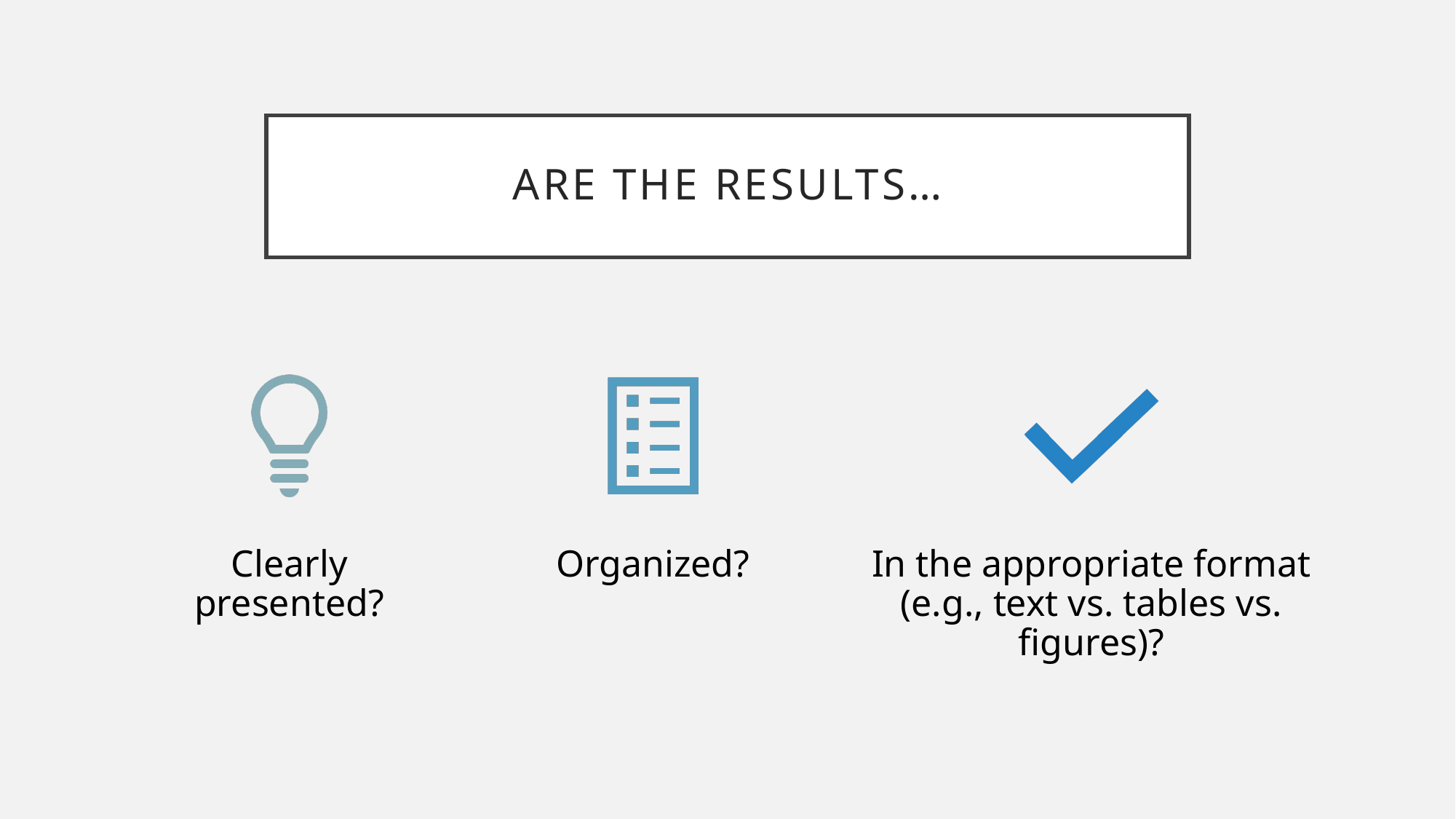

# Are the results…

## Slide 34
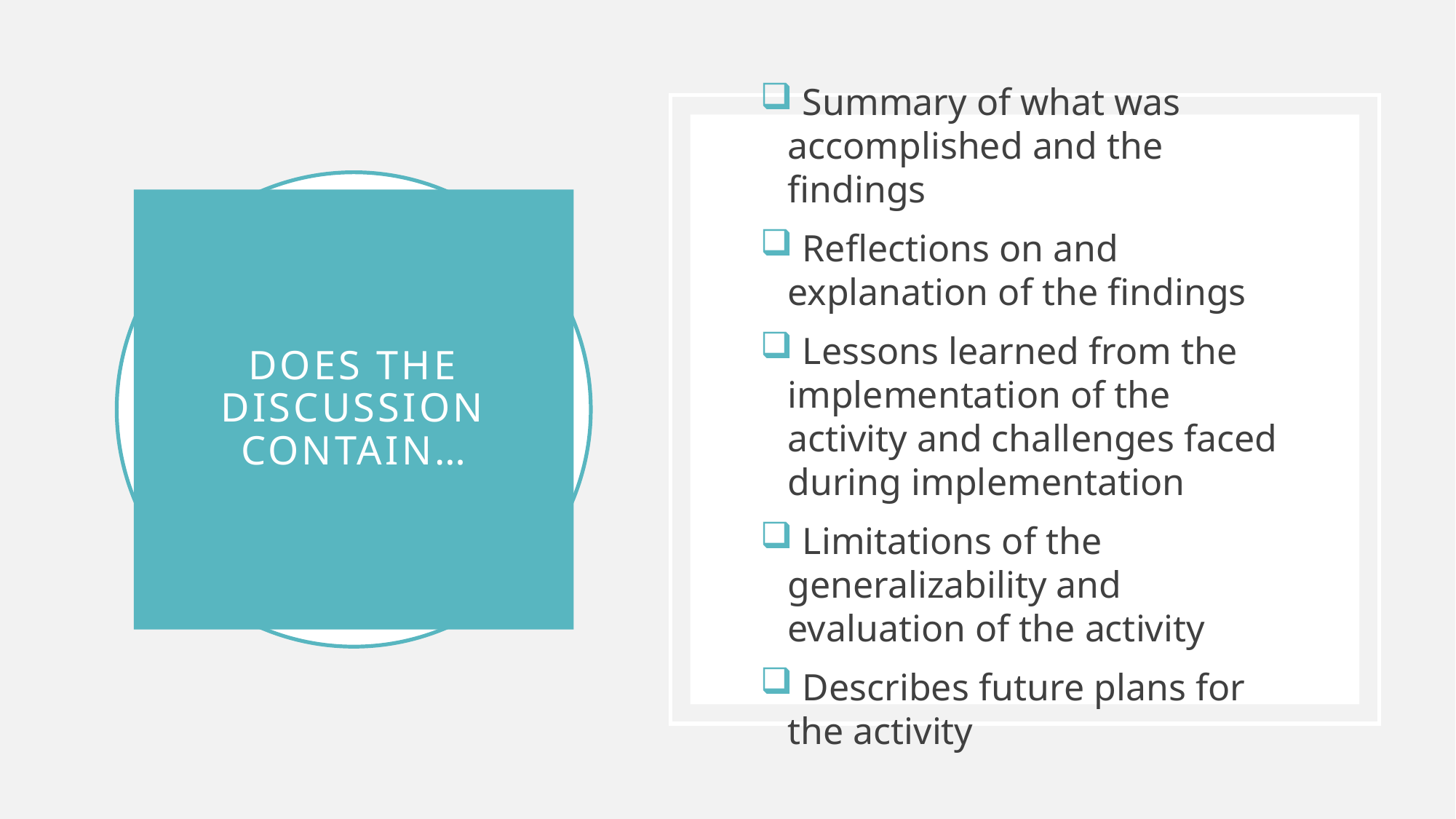

Summary of what was accomplished and the findings
 Reflections on and explanation of the findings
 Lessons learned from the implementation of the activity and challenges faced during implementation
 Limitations of the generalizability and evaluation of the activity
 Describes future plans for the activity
# Does the discussion contain…

## Slide 35
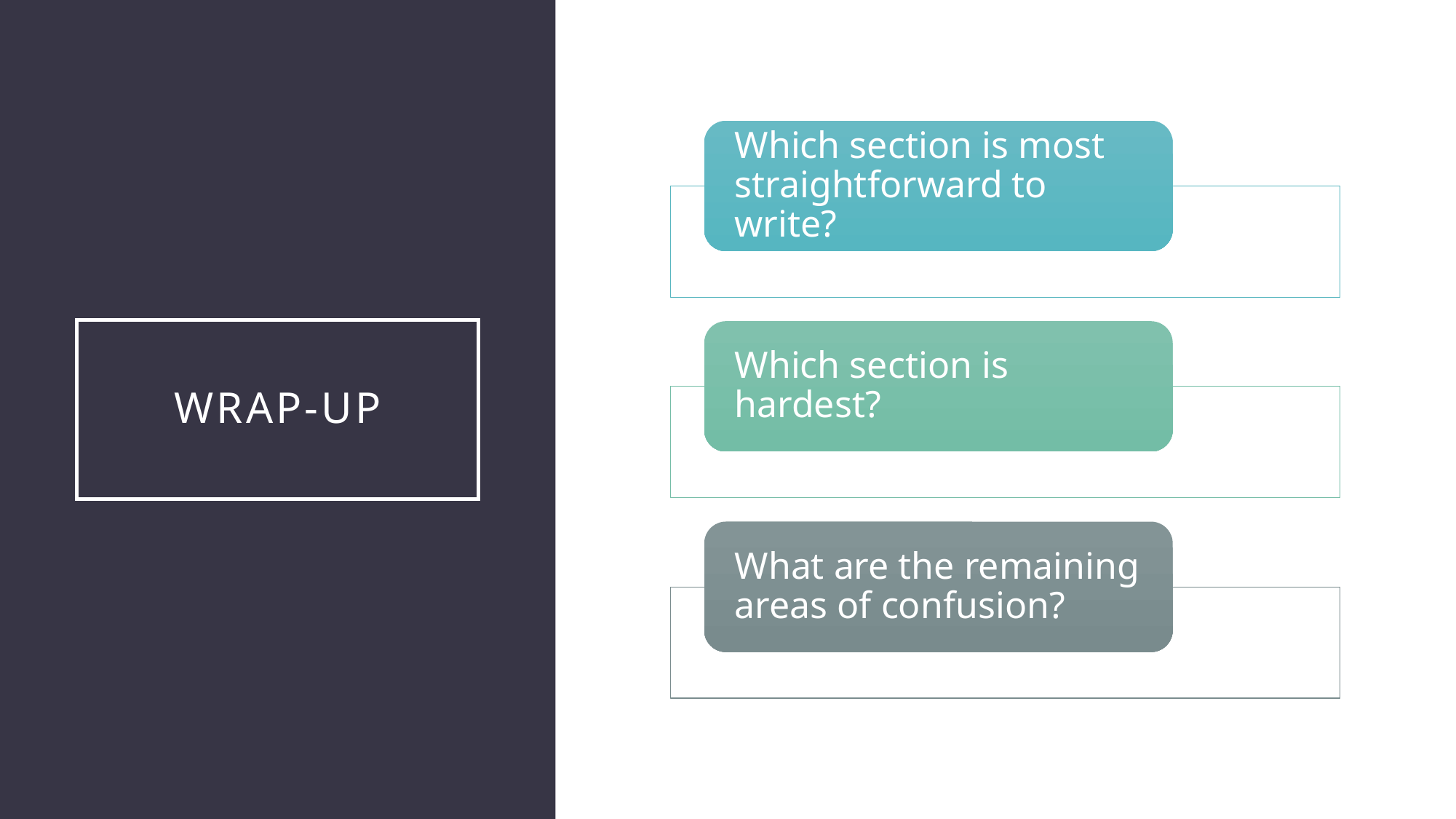

# Wrap-up
