# Supplementary material for: The MedEdPORTAL Infinity Mirror: Conducting an Interactive Workshop on How to Develop an Educational Summary Report for MedEdPORTAL
Source: MedEdPORTAL. 2021 Oct 22;17:11197. doi: 10.15766/mep_2374-8265.11197 (PMC8552417; doi:10.15766/mep_2374-8265.11197)
Supplement: Supplementary file 1 — Guidance for Facilitators.docxMEP ESR Workshop Slides.pptxEvaluating a Sample ESR.docxESR Worksheet.docxWorkshop Evaluation.docx [file mep_2374-8265.11197-s001.zip › C. Evaluating a Sample ESR.docx]

**Evaluating a Sample Educational Summary Report**

**Objectives**

| Are the objectives Specific, Measurable, Actionable, Realistic, and Time-bound (SMART)?   - Yes! - Kinda sorta - No   How would you revise the objectives to be SMART-er? ___________________________  ______________________________________________________________________ |
| --- |

**Introduction**

| Which of the following are present?   - Statement of the problem at hand - Description of the literature gap   - Have they mentioned whether there are similar MedEdPORTAL publications?   - Have they explained why other publications may fall short OR how this submission uniquely addresses the problem? - Statement of purpose   - Does it mention the learner audience?   - Does it reference the teaching approach?   How would you revise the introduction? ______________________________________  ______________________________________________________________________  ______________________________________________________________________ |
| --- |

**Methods**

| Which of the following are present?   - Description of learners / participants - Description of the curricular setting for the activity - Listing of resources needed to implement the activity - Description of teaching (or assessment) methods - Description of the evaluation approach   How would you revise the methods section? What additional details are needed to allow others to replicate the activity? ____________________________________________  ______________________________________________________________________  ______________________________________________________________________ |
| --- |

**Results**

| Are the results…   - Clearly presented? - Organized? - In the appropriate format (e.g., text vs. tables vs. figures)?   How would you revise the results section? ___________________________________  ______________________________________________________________________  ______________________________________________________________________  ***Bonus**: What other evaluation approaches would you have considered for this activity? ______________________________________________________________________  ______________________________________________________________________  ______________________________________________________________________ |
| --- |

**Discussion**

| Which of the following are present?   - Summary of what was accomplished and the findings - Reflections on and explanation of the findings - Lessons learned from the implementation of the activity - Limitations of the generalizability and evaluation of the activity - Describes future plans for the activity   How would you revise the discussion section? _________________________________  ______________________________________________________________________  ______________________________________________________________________  ***Gut check**: Does the discussion section demonstrate a scholarly reflection on the activity?   - Yes - No |
| --- |
