# Supplementary material for: The MedEdPORTAL Infinity Mirror: Conducting an Interactive Workshop on How to Develop an Educational Summary Report for MedEdPORTAL
Source: MedEdPORTAL. 2021 Oct 22;17:11197. doi: 10.15766/mep_2374-8265.11197 (PMC8552417; doi:10.15766/mep_2374-8265.11197)
Supplement: Supplementary file 1 — Guidance for Facilitators.docxMEP ESR Workshop Slides.pptxEvaluating a Sample ESR.docxESR Worksheet.docxWorkshop Evaluation.docx [file mep_2374-8265.11197-s001.zip › D. ESR Worksheet.docx]

**Educational Summary Report Worksheet**

**Objectives** – In a Specific, Measurable, Actionable, Realistic, and Timely (SMART) manner, list what learners will achieve during this session. Verb examples include: List, Describe, Apply, Analyze (but not Understand, Know, or Recognize).

| By the end of the session, learners will be able to:   1. _________________________________________________________________ 2. _________________________________________________________________ 3. _________________________________________________________________ |
| --- |

**Introduction** – Build an argument for the content being taught and/or methodology used.

| - The clinical problem is ________________________________________________ - The educational gap is that ____________________________________________ - The literature has shown that __________________________________________ but what is still missing is that _________________________________________ Why our work is unique / additive to the literature is because ________________ |
| --- |

**Methods** – Describe the implementation with sufficient detail that another faculty member could replicate it.

| - The context (e.g., course, teaching setting) in which we implemented our work was ______________________________________________________________ - The instructional methods we used were _________________________________ _________________________________________________________________ - We needed the following resources (personnel, physical space, materials) to implement the work _________________________________________________ - The documents (handouts, facilitator’s guide, slides) needed to carry out the teaching activity were:  1. ___________________________________________ 2. ___________________________________________ 3. ___________________________________________ 4. ___________________________________________ 5. ___________________________________________  - We assessed the effectiveness of our activity by __________________________ |
| --- |

**Results** – Provide evidence that learners achieved the objectives and other measures of success.

| The number of participants was ___________________________________________  Our results show that ___________________________________________________ |
| --- |

**Discussion** – Summarize the value of the session, reflect on limitations and lessons learned, and suggest future directions.

| - We developed an activity and demonstrated that __________________________ __________________________________________________________________ - In the process we learned _____________________________________________ - Challenges we faced included __________________________________________ - Our limitations include _______________________________________________ - Next steps for our work include ________________________________________ |
| --- |
