# Supplementary material for: The MedEdPORTAL Infinity Mirror: Conducting an Interactive Workshop on How to Develop an Educational Summary Report for MedEdPORTAL
Source: MedEdPORTAL. 2021 Oct 22;17:11197. doi: 10.15766/mep_2374-8265.11197 (PMC8552417; doi:10.15766/mep_2374-8265.11197)
Supplement: Supplementary file 1 — Guidance for Facilitators.docxMEP ESR Workshop Slides.pptxEvaluating a Sample ESR.docxESR Worksheet.docxWorkshop Evaluation.docx [file mep_2374-8265.11197-s001.zip › E. Workshop Evaluation.docx]

**Workshop Evaluation**

(This is the actual evaluation used in the workshop.)

|  | Strongly disagree | Disagree | Neither Agree nor Disagree | Agree | Strongly Agree |
| --- | --- | --- | --- | --- | --- |
| The content of the workshop met the description in the program. |  |  |  |  |  |
| The handout materials were clearly written and relevant. |  |  |  |  |  |
| The workshop leaders were knowledgeable and helpful. |  |  |  |  |  |
| The material presented was excellent in preparing me to use the knowledge and skills in my academic work. |  |  |  |  |  |
| The workshop presentation style was conducive to learning. |  |  |  |  |  |
| I would recommend this workshop to a colleague. |  |  |  |  |  |

**Comments:**

|  |
| --- |

**Workshop Evaluation**

(This is the evaluation we would recommend for future workshop facilitators.)

As a result of my participation in this workshop, I am able to:

|  | Strongly disagree | Disagree | Neither Agree nor Disagree | Agree | Strongly Agree |
| --- | --- | --- | --- | --- | --- |
| Compare the Educational Summary Report (ESR) in *MedEdPORTAL* with a traditional manuscript in medical education journals. |  |  |  |  |  |
| Describe how each part of a manuscript adheres to standards for scholarship. |  |  |  |  |  |
| Identify potential pitfalls in each section of an ESR. |  |  |  |  |  |
| Describe best writing practices in developing an ESR. |  |  |  |  |  |
| Draft a manuscript for submission to *MedEdPORTAL*. |  |  |  |  |  |

**Name 1 aspect of the workshop that was valuable and 1 aspect that could have been better:**

|  |
| --- |
