# Supplementary material for: The MedEdPORTAL Infinity Mirror: Conducting an Interactive Workshop on How to Develop an Educational Summary Report for MedEdPORTAL
Source: MedEdPORTAL. 2021 Oct 22;17:11197. doi: 10.15766/mep_2374-8265.11197 (PMC8552417; doi:10.15766/mep_2374-8265.11197)
Supplement: Supplementary file 1 — Guidance for Facilitators.docxMEP ESR Workshop Slides.pptxEvaluating a Sample ESR.docxESR Worksheet.docxWorkshop Evaluation.docx [file mep_2374-8265.11197-s001.zip › A. Guidance for Facilitators.docx]

**Guidance for Facilitators**

**Preparation**

- Outline for a 90-minute workshop
  - Review of ESR sections – 20 min
  - Read a sample ESR – 5 min
  - Review ESR as small groups – 25 min
  - Interactive report-out of ESR feedback based on worksheet – 20 min
  - Wrap-up and FAQ – 10 min
- Materials needed (live version)
  - Ideally 2 facilitators: 1 for the didactic, 1 as a helper/scribe/timekeeper
  - Tables with 6-8 people each
  - At least 5 flip charts, pre-scripted as shown in photos at the end of the document
  - Enough markers for each table
  - Slides for workshop
  - “Evaluating a Sample ESR” worksheet
  - Double-sided copies of ESR for review
  - Slide advancer
- Materials needed (virtual version)
  - Ideally 2 facilitators: 1 for the didactic, 1 as a timekeeper/technical support for creating and managing breakout groups
  - Slides for workshop for the facilitator who will need permission to share their screen
  - ESR for review in a Word document or PDF, emailed in advance or distributed via virtual chat
  - “Evaluating a Sample ESR” worksheet, emailed in advance or distributed via virtual chat
  - Pre-populated Google Docs for small group breakouts, using the pre-scripted flip charts as a scaffold

**Choosing an ESR for review**

If you choose to identify a draft ESR (one that has not been submitted), keep in mind that there were challenges we uncovered in running this workshop, as described in the ESR. If you decide to take this route:

- First reach out to key leaders within the target organization or local institution.
- Send emails to recruit to prospective MEP authors to submit ESRs for review.
  - - Connect with the prospective authors to offer guidance and resources
    - Set expectations for having a draft ESR, ideally 3 weeks prior to workshop
    - Provide feedback to authors 2 weeks in advance of workshop regarding missing elements (e.g., more references, including structured abstract, alignment with worksheet)
    - Finalize draft ESR for distribution

We do recommend instead that you choose a published *MedEdPORTAL* ESR, using the following guidelines:

- - Select a MedEdPORTAL publication from the website with a publication date between July 2016 to the present, which corresponds with implementation of the ESR (previously, submissions only required instructor’s guides)
  - Consider your target learner audience’s specialty or background in your selection of publication topics.
  - Aim for a publication that is reasonable in size, including that it only has several appendices, encompasses a discrete education activity < 90 minutes, and does not contain multiple parts (e.g., several simulation cases).
  - Some example publications to consider (target audience-dependent) that encompass these suggestions:

Dai JC, Ahn JS, Cannon ST, et al. Acute ischemic priapism management: an educational and simulation curriculum. *MedEdPORTAL*. 2018;14:10731.

<https://www.mededportal.org/doi/10.15766/mep_2374-8265.10731>,

Berrocal Y, Fisher J, Regan J, Christison AL. Dehydration: a multidisciplinary case-based discussion for first-year medical students. MedEdPORTAL. 2018;14:10725.

<https://www.mededportal.org/doi/10.15766/mep_2374-8265.10725>,

Niedermier J, Kasick D. Maintaining personal safety: understanding and addressing aggression and violence in the health care setting. MedEdPORTAL. 2018;14:10722.

<https://www.mededportal.org/doi/10.15766/mep_2374-8265.10722>.

**Facilitation tips**

As the activity is intended to be an interactive workshop, encourage questions, especially during the didactic portion to break up the flow of the didactic. Provide context on the ESR you are using for the review activity. For instance, clarify that the draft ESR has not been submitted formally to MedEdPORTAL, if applicable, or describe why you chose the published ESR being used for review. For live iterations of the workshop, walk around the room during the small group exercise to listen in and provide guidance. Most of all, keep track of time, as there are several elements of this workshop that can take an unpredictable amount of time.

Managing the report-out can be challenging for any workshop after returning from breakout groups. Participants may desire to narrate how the discussion evolved rather than focusing on concrete answers to the questions, and additionally, having a platform to speak takes some of the control of timing out of the hands of the facilitators. Begin with a reminder that you do not need them to summarize what was discussed, that you will moderate heavily to give other groups a chance to report out and may cut them off.

Also consider asking specific questions for particular manuscript aspects:

- - Objectives – ask how they would revise one of the objectives
  - Introduction – ask them to tell you what was the “Problem, Gap, Purpose”
  - Methods – ask them what was missing or required more clarification
  - Results – ask how they presented the data and how it could be improved
  - Discussion – ask which of the 5 paragraphs needed more detail

Lastly, handling questions requires some skill and intentionality. For tangential questions, if they cannot be answered quickly, delegate the questions for later or offer to answer the question one-on-one after the workshop. For questions that relate very specifically to someone’s project, try to generalize the situation such that it applies to other scenarios.

**Pre-scripted flip charts for 5 breakout groups in a live workshop format**


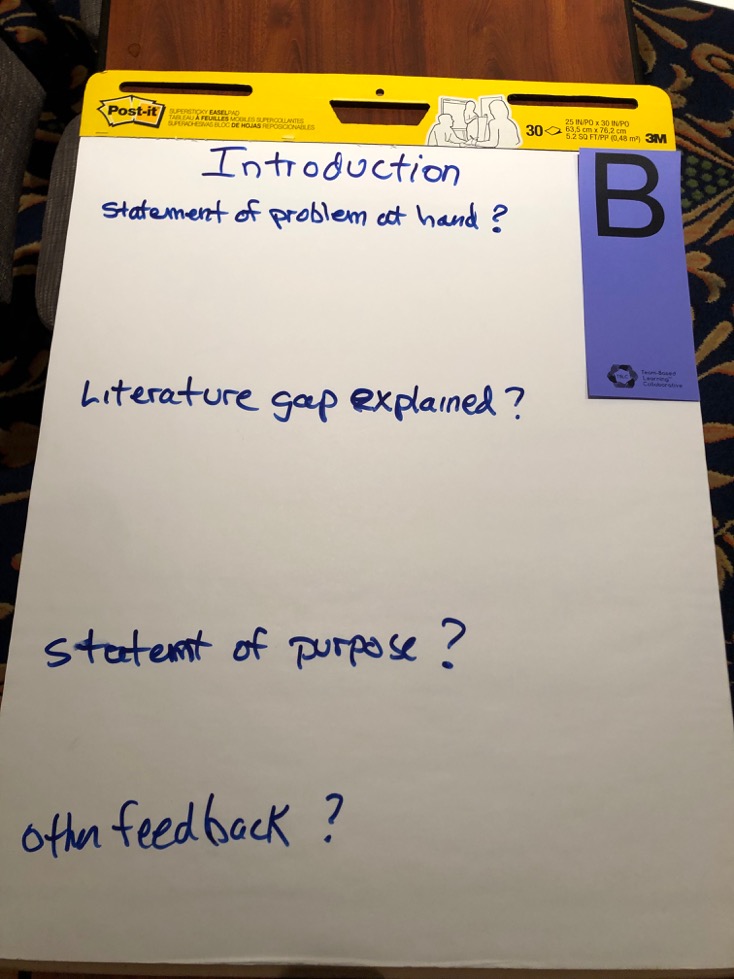

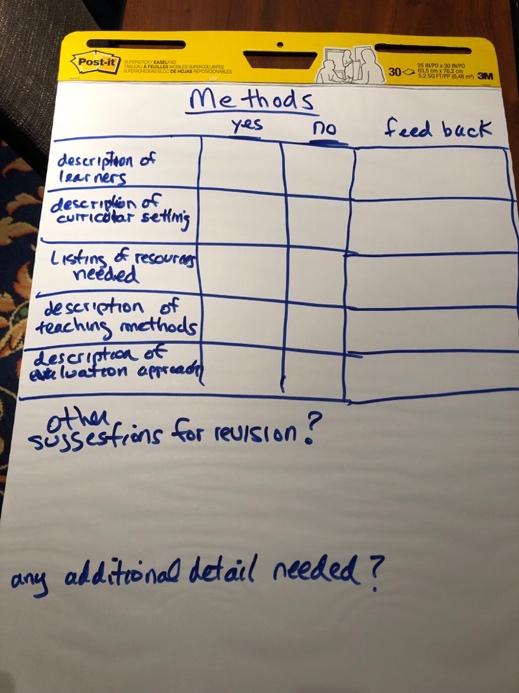


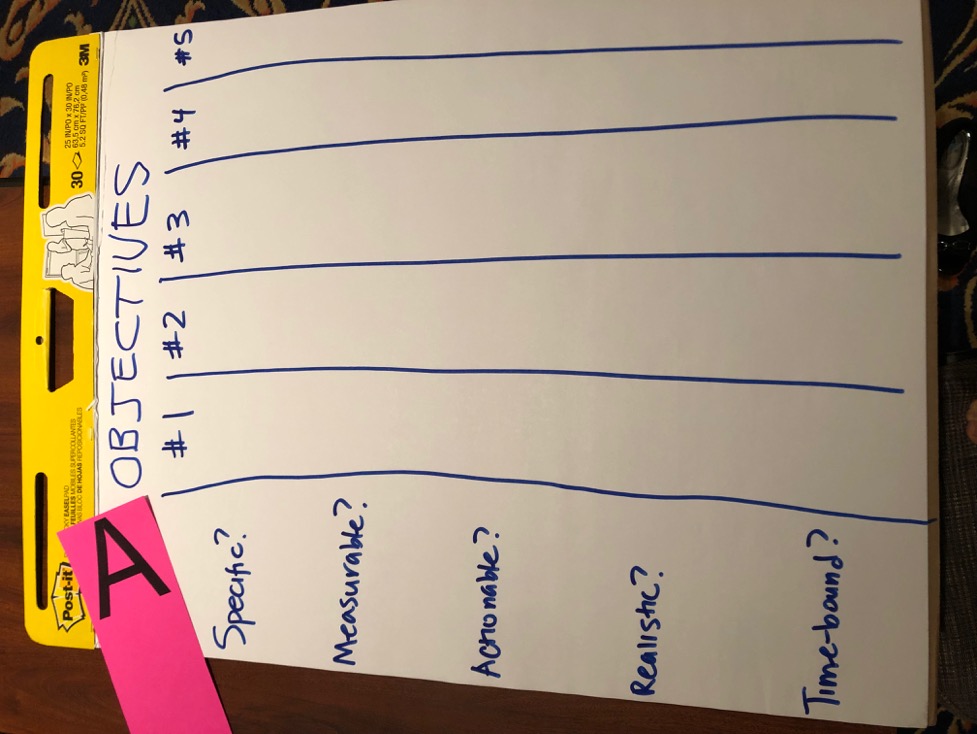


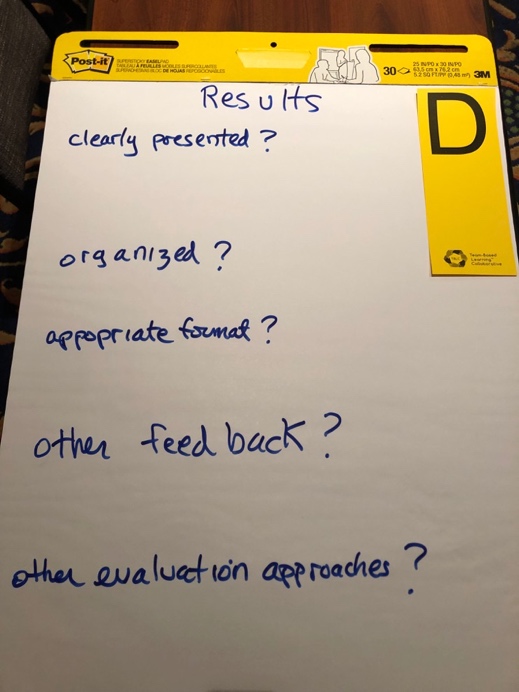

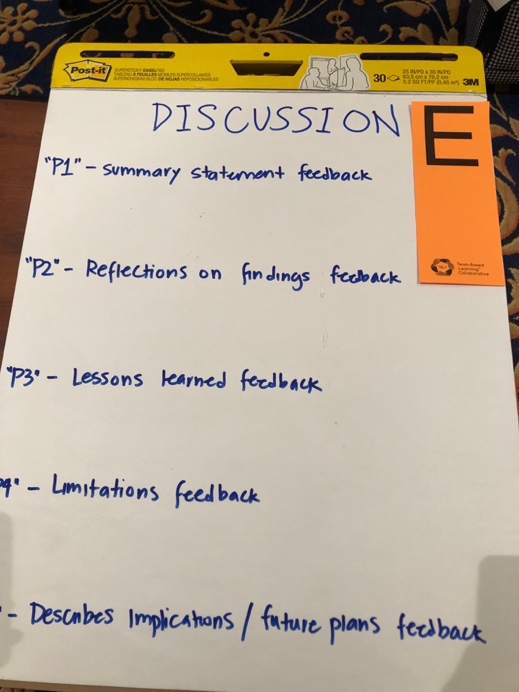


Photos: Author owned.
